# A Shape‐Adaptive, Performance‐Programmable, Self‐Healable and On‐Demand Destructible Robotic Skin via Self‐Strengthening Dynamic Silicone

**DOI:** 10.1002/advs.202508823

**Published:** 2025-07-26

**Authors:** Wusha Miao, Lara S. Laamari, Jing Yu, Sanjay Schreiber, Lukas Heer, Jiacheng Cui, Jiayuan Huang, Hedan Bai

**Affiliations:** ^1^ Laboratory of Robotic Materials Department of Materials ETH Zürich Hönggerbergring 64 Zürich 8093 Switzerland; ^2^ Key Laboratory of Silkworm and Bee Resource Utilization and Innovation of Zhejiang Province Institute of Applied Bioresource Research College of Animal Science Zhejiang University Hangzhou 310058 P. R. China; ^3^ State Key Laboratory of High‐performance Precision Manufacturing Dalian University of Technology Dalian Liaoning 116024 P. R. China

**Keywords:** dynamic siloxane bonds, Omni‐adaptive robotic skin, reconfiguration

## Abstract

The ability of robotic devices to adapt like living organisms to their environment is fundamental to achieving physical intelligence. Robotic skin that modulates its morphology, function, and lifetime in situ can approach the intelligent tactile senses in organisms. Despite the recent advances in each of these adaptive functions, robotic skin that is adaptive in all these aspects remains elusive. In this work, an omni‐adaptive capacitive pressure sensor based on dynamic silicone materials is presented, which can undergo distinct inter‐ and intra‐chain bond exchange pathways. Utilizing a superbase phosphazene catalyst, silanolate species are generated that can attack siloxane bonds within the same chain (intra‐chain) and between different chains (inter‐chain), which enables self‐healable and shape reconfigurable performance. Notably, intra‐chain exchanges lead to the formation of volatile cyclic siloxanes that can escape from the network, allowing for controlled programmability of the polymer network and corresponding mechanical properties. Furthermore, by shifting the reaction equilibrium toward more cyclic siloxanes generation, this demonstrates on‐demand material degradation. Leveraging this dynamic framework, the omni‐adaptive robotic skin exhibits shape‐adaptation, performance‐programmability, self‐healing, and on‐demand destruction, which promises a wide range of applications from wearable devices, haptic feedback for MIS practice to self‐healing and on‐demand destructible robotic skin.

## Graphical abstract

Enabled by the distinctive inter‐ and intra‐chain siloxane exchange in commercially available silicones as the dielectric layer, the omni‐adaptive robotic skin (OmniAdapt) integrates multiple advanced functionalities: programmable sensing performance via the self‐stiffening dielectric material, application as BPM sensor, shape adaptivity for electronic skin (E‐skin), self‐healing capability and on‐demand destructibility.

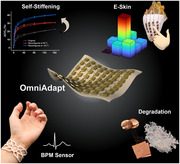

## 1 Introduction

Adaptability, one of the most distinct features of biology, is the foundation of physical intelligence.^[^ ^1^, ^2^ ^]^ Robotic skin that adapts its shape, performance, and lifetime to the environment can bridge the gap between synthetic devices and lifelike tactile senses.^[^ ^3^, ^4^, ^5^, ^6^, ^7^, ^8^, ^9^ ^]^ Significant progress has been made in the field of robotic skin through bottom‐up material innovations, aimed at adaptivity.^[^ ^10^, ^11^, ^12^, ^13^ ^]^ For example, Jeong et al. developed an adaptive robotic skin utilizing the solid‐liquid phase transition of gallium microgranules embedded in an elastomeric matrix.^[^ ^14^ ^]^ This design allows for tunable pressure sensing performance, with a high sensitivity of 16.97 kPa^−1^ in the soft mode and a broad detection range up to 1.45 MPa in the rigid mode. For lifetime programming, self‐healing tactile sensing has also been achieved with material advances that self‐heals through either intrinsic dynamic bonds or extrinsic particles or networks.^[^ ^15^ ^]^ By embedding conductive microparticles within a supramolecular polymer matrix, both mechanical and electrical self‐repair are achieved via dynamic hydrogen bonding and reconnection between adjacent conductive pathways.^[^ ^16^ ^]^ Moreover, when it comes to the end of life, on‐demand destruction^[^ ^17^ ^]^ could ensure data security and bio‐based sensor degradation^[^ ^18^, ^19^ ^]^ demonstrates leaps forward toward sustainability.^[^ ^20^, ^21^ ^]^

These advancements underscore the important role of advanced materials in the evolution of robotic skins.^[^ ^22^ ^]^ An interesting question to ask is if there exists a robotic material that could embed all the above adaptive functions. Among emerging materials, dynamic covalent polymers have shown promise due to their intrinsic adaptability including self‐healing, chemical recyclability, and shape reconfigurability.^[^ ^23^, ^24^, ^25^ ^]^ These properties arise from the dynamic bonds, which undergo associative or dissociative exchange reactions.^[^ ^26^ ^]^ For instance, fully recyclable and self‐healable electronic skins were fabricated from dynamic polyimine materials, demonstrating self‐healing and recyclability from dynamic amine‐aldehyde bond exchange.^[^ ^27^ ^]^ In robotic applications, the viscoelasticity inherent to reversible bonds can introduce sensing drift. To address this, Shepherd et al. implemented a wavy structural design to exploit compliance and achieve fully elastic response in self‐healing optical sensors enabled by dynamic hydrogen and disulfide bonding.^[^ ^15^ ^]^ Nevertheless, the key limitation of dynamic polymer networks lies in their unchanged network topology before and after bond exchange due to the intrinsic bond exchange reversibility,^[^ ^28^ ^]^ therefore limiting programmability of mechanical and sensing properties. Consequently, robotic skins fabricated from such materials typically lack reconfigurable and programmable performance. Furthermore, realizing an adaptive robotic skin that seamlessly integrates shape adaptability, mechanical reconfiguration, self‐healing, and on‐demand degradation has yet to be achieved.

To overcome these challenges, we introduce commercially available dynamic silicone materials toward the omni‐adaptive robotic skin (we abbreviate for “OmniAdapt”), enabling all shape‐adaptation, performance‐programmability, self‐healing and on‐demand destruction in a single robotic material. From the material perspective, dynamic siloxane equilibrium can be traced back to 1950s, when Tobolsky observed the stress relaxation in silicone rubbers and attributed it to the ionic interchanges of siloxane bonds triggered by the residual acid catalyst.^[^ ^29^ ^]^ This was further verified by Grubb and colleagues.^[^ ^30^ ^]^ Despite these early insights, dynamic siloxane equilibration was largely overlooked for several decades. It was not until 2012 that McCarthy et al. re‐investigated the phenomenon, demonstrating anionic siloxane exchange in the presence of tetramethylammonium silanolate, which was utilized for self‐healing.^[^ ^31^ ^]^ Since then, dynamic siloxane bond exchange has been systematically explored within suitable catalysts, establishing the foundation in adaptive materials.^[^ ^32^, ^33^ ^]^ Herein, by employing a superbase phosphazene catalyst (P₄‐*^t^* Bu), silanolate intermediates are generated that can participate in either intra‐chain or inter‐chain siloxane bond exchange, enabling both shape reconfiguration and self‐healing. Uniquely, the intra‐chain exchange results in the formation of volatile cyclic siloxanes, which can escape from the system, inducing relative fillers contents increase and correspondingly enhance mechanical strength. This dual‐pathway mechanism is compatible with commercially available silicone materials, significantly expanding their applicability. We demonstrate the implementation of OmniAdapt by fabricating capacitive pressure sensors in which the dynamic silicone network functions as the dielectric layer. Programmable and reconfigurable sensing performance, with sensitivity increasing from 1.68 to 4.67 kPa^−1^ was achieved due to the mechanical tunability via the dual siloxane bonds exchange pathways. Besides, the sensors exhibit unique self‐healing behavior, restoring functionality, while simultaneously reconfiguring their sensing performance, compared to the pristine state. Shape reconfiguration allows the sensor to conform to complex geometries, such as a heart model with intricate topologies, illustrating potential for haptic feedback applications in minimally invasive surgery (MIS). Moreover, the sensor can undergo degradation under higher phosphazene catalyst loading by shifting the dynamic exchange equilibrium toward the formation of cyclic monomers that escape from network, leading to material disassembly. Overall, the omni‐adapt skin enables a wide range of applications: wearable devices, haptic feedback for MIS practice, self‐healing and on‐demand destructible robotic skin. Our findings establish a versatile and scalable framework for dynamic covalent polymers in robotic skin applications.

## 2 Results and Discussion

### 2.1 Overview of Dynamic Silicones Toward Programmable Robotic Skin that Adapts and Reconfigures

**Figure 1:**
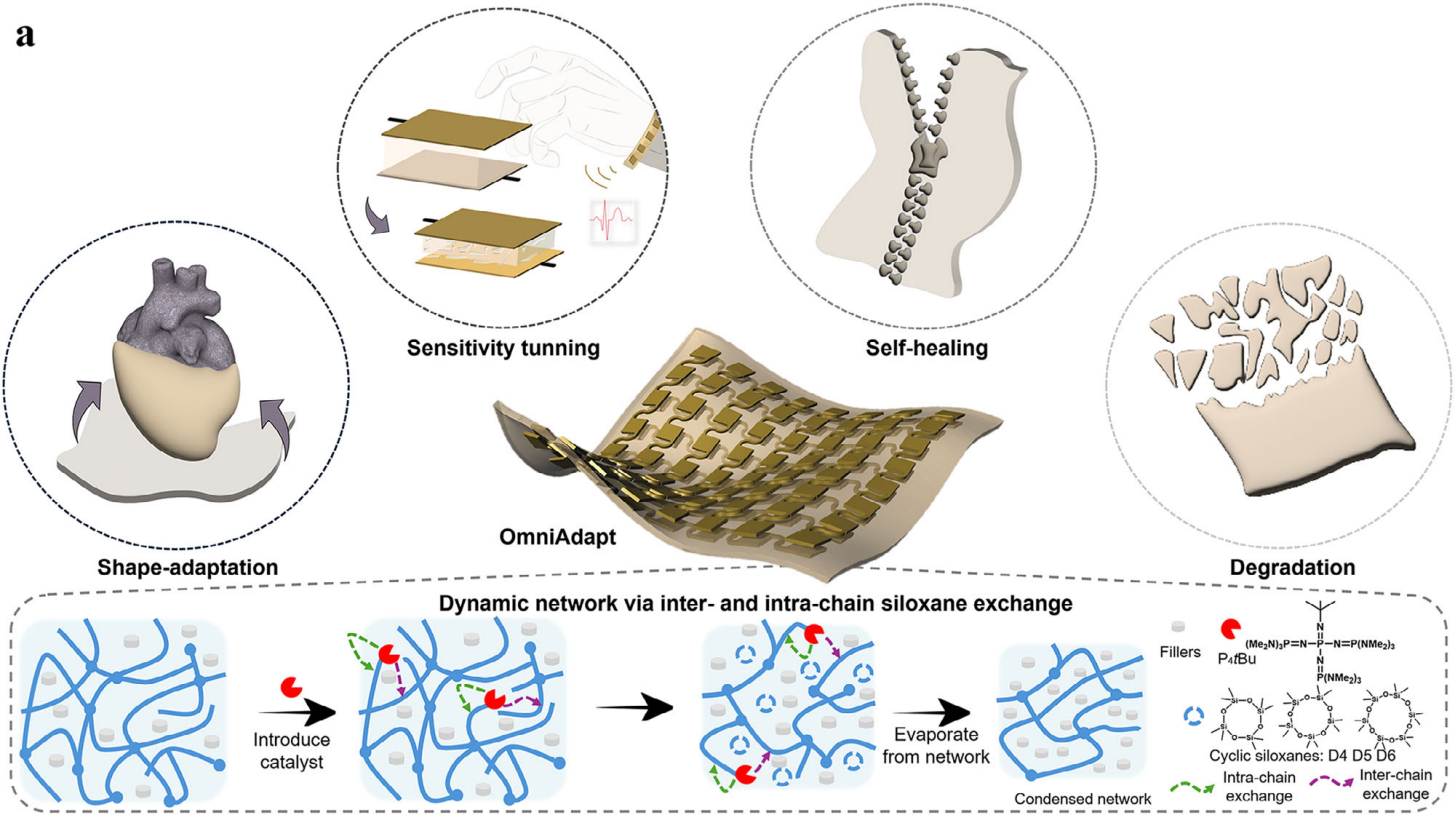
Overview of on‐demand programmable silicones toward Omni‐Adaptive robotic skin. a) Omni‐Adaptive robotic skin integrated with shape adaptability, sensitivity tuning, self‐healing, and on‐demand destruction, enabled by P₄‐*^t^* Bu triggered silanoate exchange via inter‐chain and intra‐chain pathways.

**Figure** **1**a illustrates the concept of our Omni‐Adaptive robotic skin integrated with shape adaptability, mechanical reconfiguration, self‐healing, and on‐demand destruction. In detail, we implement the OmniAdapt material as the dielectric layer in a capacitive pressure sensor. The sensor is constructed by aligning copper electrodes on either side of the dielectric film, encapsulated with thin polyimide (PI) and PDMS layers. This configuration provides a robust sensing platform where the dynamic material serves as structural and functional core. Through controlled thermal treatment, the sensor can be permanently reshaped into complex geometries, such as curved or folded configurations. This shape reconfigurability allows seamless conformability to various surfaces, making it well suited for robotic functionalities such as tactile robotic skin. Further, inter‐ and intra‐chain exchanges driven by thermal activation led to the condensation of silicone networks, which resulted in thinning and mechanical stiffening of the dielectric film. These structural changes enhance the sensor's performance by increasing sensitivity and response time, making it better suited for precise measurements such as wearable monitoring. Additionally, the dynamic network exhibits intrinsic self‐healing properties. Upon mechanical damage, the dynamic siloxane exchange led to bond formation at the damaged interfaces, enabling the closure of gaps or to reattach cut‐off components. Finally, the degradation of the material at the sensor end of life is possible by thermal treatments and increased catalyst concentration, offering secure disposal after temporary deployment.

The key challenge to OmniAdapt lies in designing a substrate material that fulfills these adaptations simultaneously, functions that usually require elegant network design. In this work, we aim to develop a method widely applicable to commercial silicone, which typically incorporates silica fillers to tune mechanical properties. Previous work has demonstrated the pure silicone elastomer can be reprocessed via P₄‐*^t^* Bu triggered dynamic siloxane bonds exchange.^[^ ^34^ ^]^ However, mechanical degradation was observed upon recycling in the silica‐free PDMS network, which is not aligned with the objectives of our Omni‐Adapt design. Herein, our approach leverages a dynamic network based on commercially available silicone elastomers typically containing silica fillers. Utilizing the phosphazene catalyst P₄‐*^t^* Bu, which facilitates both inter‐ and intrachain siloxane bond exchange, we show that network isomerization and tunable mechanical behavior are uniquely enabled by the presence of silica fillers, commonly found in commercial formulations. We exploit P₄‐*^t^* Bu unique ability to modulate mechanical properties, shifting from soft, reconfigurable states to stiffened or even disassembled networks depending on catalyst concentration and thermal input. This unique mechanism offers specific programmability in a system based on commercially available polydimethylsiloxanes (PDMS) composites with fillers. These materials are widely used for their broad industrial applications, favorable thermal and mechanical properties, making them excellent materials for soft robotics.

### 2.2 Inter‐Chain and Intra‐Chain Exchange Mechanism at the Molecular and Network Levels

To explore the dual siloxane exchange pathways under P₄‐*^t^* Bu, we designed a model experiment (Figure S1, Supporting Information) with two objectives: 1) to verify whether P_4_‐^t^Bu‐activated silanolates can trigger intra‐chain exchange of back‐biting reactions in addition to the well‐studied inter‐chain pathway;^[^ ^34^ ^]^ 2) to identify specific products generated through intra‐chain exchange. Details of the formulation and procedures are provided in the Experimental Section. We note that the inter‐chain exchange occurs when silanolates attack siloxane bonds between different polymer chains. In contrast, intra‐chain exchange involves silanolates attacking siloxane bonds within the same polymer chain, potentially yielding cyclic siloxanes. The mechanism is characterized by monitoring the peak change in ^29^Si‐NMR analysis. The chemical shift of silicon atoms depends on their bonding environment, especially chemical environment around the silicon atom and the siloxane ring size or linearity. In our model experiment results, all these compounds feature D^2^ units (Si bonded to two O and two CH₃). As the ring size increases (from D₄ to D₆), the ^2^⁹Si chemical shift becomes more negative (upfield). This is due to reduced ring strain and increased Si‐O‐Si bond angles, which influence the electronic environment around silicon. Linear PDMS is the most upfield. As shown in Figure S2a (Supporting Information), the control sample without P₄‐*^t^* Bu exhibits a single resonance at ‐21.8 ppm, corresponding to linear siloxane bonds. Upon the addition of P₄‐*^t^* Bu and annealed at 25 °C for 3 h, new signals appear at ‐18.9 and ‐21.3 ppm, which are assigned to octamethylcyclotetrasiloxane (D₄) and decamethylcyclopentasiloxane (D₅) cyclic siloxanes, respectively. A further shift from ‐21.8 ppm of linear siloxane bonds, observed at ‐21.7 ppm, is attributed to dodecamethylcyclohexasiloxane (D₆) formation and mixed with linear siloxane bonds. The intensities of these peaks increase with the additive concentration, confirming that P₄‐*^t^* Bu indeed initiate intra‐chain exchange and facilitates the generation of cyclic siloxanes (D_4_, D_5_ and D_6_), beyond inter‐chain exchange previously thoroughly studied. We further observed that elevated temperatures accelerated this exchange process. Figure S2b (Supporting Information) shows that higher annealing temperatures resulted in greater peak intensities of D₄, D₅, and D₆, indicating faster siloxane exchanges and more cyclic monomers formation.

**Figure 2:**
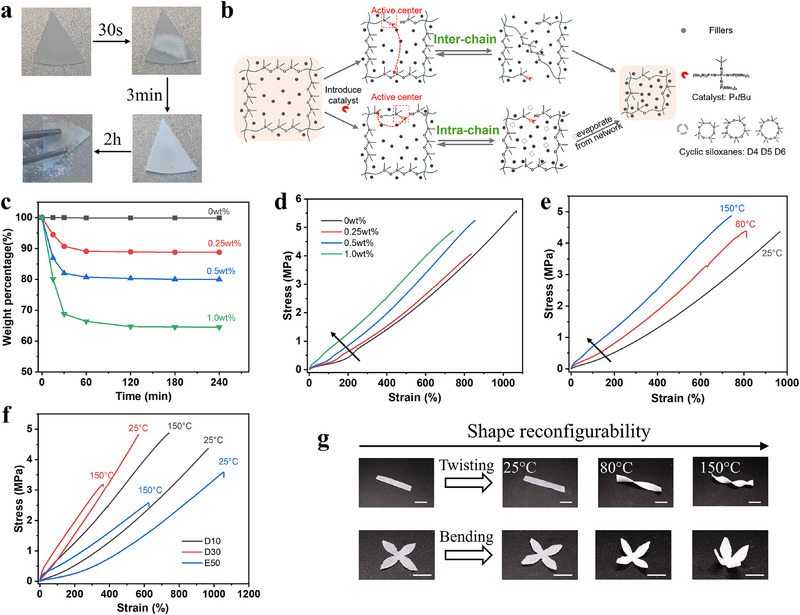
On‐demand dynamic silicones with distinctive inter‐ and intra‐chain exchange pathways. a) Macroscopic change in D10 samples with 1 wt.% P₄‐*^t^* Bu under 150 °C; b) Schematic illustration of distinctive siloxane exchange at the network level; c) Weight change from cyclic monomers (D_4_, D_5_ and D_6_) with time containing different amounts of P₄‐*^t^* Bu at 150 °C for D10; d) Stress‐strain curves of D10 samples with different P₄‐*^t^* Bu loading; e) Stress‐strain curves of D10 samples within different temperature thermal annealed under 1wt.% P₄‐*^t^* Bu; f) Stress‐strain curves of different silicones before and after thermal treatment. All sample contained 1wt.% P₄‐*^t^* Bu; g) Shape reconfiguration of D10 within 1 wt.% P₄‐*^t^* Bu at different temperature (all scale bars: 10 mm).

Having elucidated the dual‐pathway exchange mechanism at the molecular level, we next investigate its effect at the network level using commercially available silicones. It is worth noting that the phosphazene base, P₄‐*^t^* Bu, can interfere with platinum‐catalyzed silicone crosslinking. Therefore, to avoid network inhibition, the catalyst was introduced via post‐swollen into cured silicones. Full details are provided in the Experimental Section. We began with Dragon Skin 10 (D10) silicone as the model network. Upon incorporating 1 wt.% P₄‐*^t^* Bu and annealing at 150 °C, we observed a striking macroscopic change: within 30 s, the sample partially turned opaque, which propagated throughout the material over 3 min (**Figure** **2**a). After 2 h of annealing, we recorded a weight loss of up to 35.5%, which could be attributed to the evaporation of cyclic siloxanes (D₄, D₅, D₆) formed via intra‐chain exchange. Concurrently, white particulate powder detached from the network, which is unexpected. The controlled sample without catalyst exhibits no macroscopic change and weight loss (Figure S3, Supporting Information). To identify these particles, thermogravimetric analysis (TGA) was conducted, revealing a slight weight loss from polymer thermal degradation (Figure S4, Supporting Information). The residual mass reaches 91.3% under 700 °C, indicative of inorganic component. Subsequent energy‐dispersive X‐ray spectroscopy (EDS) confirmed the particles consisted solely Si and O (Figure S5, Supporting Information). Therefore, these white powdery particles should be silica fillers, which are commonly incorporated into commercial silicones formulations. We explain that the detachment of these fillers results from the loss of volatile cyclic siloxanes (∼35.5%), which disrupts the polymer matrix and compromises the physical encapsulation of the fillers, thereby facilitating their release. These findings demonstrate that P₄‐*^t^* Bu‐triggered dual‐pathway siloxane exchange not only enables the generation and release of cyclic siloxanes, but also induces filler removal, offering a powerful, scalable strategy for post‐synthetic polymer network reprogramming in commercial silicone networks. As schematized in Figure 2b, the phosphazene catalyst generates silanolate active species capable of attacking siloxane bonds in intra‐ and inter‐chain route. The bond exchange would enable shape reconfiguration and self‐healing ability. Interestingly, intra‐chain exchange yields volatile cyclic monomers that escape from networks. This loss of material drives the partial removal of fillers and results in a condensed, topologically altered network, offering a robust strategy for structural and mechanical tunability in commercially available silicones. Additionally, we investigated the chain mobility before and after the addition of P₄‐*^t^* Bu (Figure S6, Supporting Information). We evaluated the network sol fraction by measuring weight loss after a post‐swelling process in toluene. The control sample, without catalyst, exhibited a weight loss of ≈16 wt.%, which we attribute to the presence of soluble or un‐crosslinked chains within the network. Upon increasing the P₄‐*^t^* Bu catalyst loading, the weight loss increased progressively, reaching ∼35 wt.% at 1 wt.% catalyst concentration. We reason that during the swollen process, the solvents promote chain mobility, enabling that P₄‐*^t^* Bu could attack the siloxane polymer chain to form the active center (as illustrated in Figure 2b). This could lead to the slight decrease of crosslinking density. This observation is consistent with previously reported findings.^[^ ^34^ ^]^ To evaluate the mechanical implications of catalyst‐induced mobility, tensile testing was also performed. The results revealed only a minor reduction in mechanical strength upon P₄‐tBu addition. Together, these findings support the conclusion that P₄‐*^t^* Bu modestly enhances chain mobility.

### 2.3 Macroscopic Dynamicity and Programmability in Commercially Available Silicones

Having investigated the P₄‐*^t^* Bu‐triggered siloxane exchange mechanism through distinct intra‐ and inter‐chain pathways at the molecular and network levels, we next systematically study the resulting macroscopic dynamicity and programmability of silicone materials. As shown in Figure 2c, we quantified the weight loss associated with volatile cyclic monomer release from the D10 network under varying concentrations of P₄‐*^t^* Bu. All samples were thermally annealed at 150 °C, and the mass change over time was recorded. In the absence of P₄‐*^t^* Bu, no measurable weight loss was observed. Within P₄‐*^t^* Bu, weight loss occurred immediately and gradually increased with time. Weight percentage plateaued after 1 h, with total retention decreasing from 88.8% (0.25 wt.% P₄‐*^t^* Bu) to 64.5% (1.0 wt.% P₄‐*^t^* Bu) as catalyst concentration increased. This confirms that P₄‐*^t^* Bu is the key to siloxane dual‐pathways exchanges. In parallel, detachment of silica fillers was again observed, consistent with the proposed mechanism whereby loss of polymeric matrix disrupts the physical entrapment of the fillers. Figure S7 (Supporting Information) shows that the maximum silica loss was 4.64% at 1.0 wt.% P₄‐*^t^* Bu, significantly lower than the cyclic monomer loss (∼35.5%). This disproportionate removal results in a relative enrichment of fillers, which directly impacts mechanical behaviors. Tensile testing (Figure 2d) revealed a significant increase in Young's modulus, from 0.44 MPa (0 wt.% P₄‐*^t^* Bu) to 1.35 MPa (1.0 wt.% P₄‐*^t^* Bu). Here, we explain that the elastic modulus strengthening‐up resulted from increased relative concentration of silica fillers. FTIR analysis (Figure S8, Supporting Information) supports this interpretation, showing a decrease in PDMS characteristic absorption bands with increasing catalyst content, indicating a reduction in the siloxane matrix and an enrichment of silica components. We note that dynamic siloxane bond exchange triggered by P₄‐^t^Bu can occur in PDMS, both with and without silica fillers, enabling shape‐adaptive and self‐healing capabilities. Previous work demonstrated reprocessable silica‐free silicone elastomers with the same catalyst.^[^ ^34^ ^]^ However, mechanical degradation was observed upon recycling in the silica‐free PDMS network, which is not aligned with the objectiveness of our Omni‐Adapt design. Our focus instead is on commercially available silicone elastomers, which typically incorporate silica fillers to tune mechanical performance. Interestingly, these composite PDMS networks exhibit distinct behaviors with the same catalyst, including performance programmability and self‐strengthening. We attribute this distinction to the increasing relative concentration of silica fillers during the programming process. Specifically, at 150 °C, the weight loss of cyclic monomers in the 1 wt.% catalyst sample reaches 35.5%, significantly higher than that of the silica fillers (4.64%). This selective mass loss leads to a relative enrichment of the silica content, which contributes directly to mechanical reinforcement. These findings suggest the essential role of silica‐PDMS compositing in achieving the desired performance‐programmable and self‐strengthening characteristics. We next evaluated the effect of temperature on the dynamic behavior in samples containing 1.0 wt.% P₄‐*^t^* Bu. At 25 °C, no weight loss occurred, indicating that siloxane exchange is inactive at room temperature. At 80 °C, gradual weight loss was observed, plateauing after 3 h. At 150 °C, more significant weight loss occurred rapidly, reaching a plateau within 1 h (Figure S9, Supporting Information). We reason that two factors govern this temperature dependence: (1) the rate of dynamic bond exchange, which increases with temperature, and (2) the volatility of cyclic monomers, which enhances their escape from the network. The volatilization of D₄, D₅, and D₆ drives the intra‐chain exchange equilibrium forward, favoring further formation of cyclic species that could escape from network, which consequently lead to enhanced weight loss with higher temperature. It should be noted that the P₄‐*^t^* Bu can undergo deactivation. Previous study confirms that it can undergo partial deactivation ≈ 80–100 °C and total deactivation above 150 °C. After thermally annealed at 150 °C for 4 h, the P₄‐*^t^* Bu ‐triggered silanoates are deactivated, not capable of bond exchangeability, which implies that the dynamic network can be reverted into permanent work. This offers the opportunity to on‐demand control dynamicity by temperature in our silicone networks. Besides, filler detachment increased with temperature, from 2.65 wt.% at 80 °C to 4.64 wt.% at 150 °C, while no filler removal was detected at 25 °C (Figure S10, Supporting Information). The Young's modulus increased with temperature, from 0.35 MPa (25 °C) to 1.35 MPa (150 °C) in Figure 2e, mirroring the trend observed with increasing P₄‐*^t^* Bu loading. FTIR spectra (Figure S11, Supporting Information) again confirmed the mechanical strength‐up results from relatively higher amount of silica fillers by exhibiting the significant decrease of PDMS characteristic absorption bands. This temperature‐controlled programmability presents exciting opportunities in reconfigurable robotic systems with tunable actuation or sensing functions. To demonstrate the broader applicability, we extended this approach to other commercial silicones, including Dragon Skin 30 (D30) and Ecoflex 50 (E50). In Figure S12 (Supporting Information), samples containing 1.0 wt.% P₄‐*^t^* Bu exhibited significant weight loss at 150 °C, reaching 76.3% for D30 and 50.6% for E50. The differences are attributed to the intrinsic network stiffness and chain mobility: D30 is stiffer than D10, resulting in reduced exchange kinetics, whereas E50 is more compliant, facilitating faster dynamic exchange. Corresponding filler losses were 2.12 wt.% for D30 and 9.26 wt.% for E50 (Figure S13, Supporting Information). All programmed silicones showed a mechanical stiffening effect with reduction in elongation (Figure 2f), resulted from relative higher content of silica fillers, which confirmed by PDMS characteristic absorption bands decreasing in FTIR (Figure S14, Supporting Information). We note that the increasing concentration of silica fillers imposes geometric and interfacial constraints on the polymer chains, thereby restricting their mobility through physical confinement. Herein, P₄‐*^t^* Bu‐triggered dual‐pathway siloxane exchange provides a universal platform for topological programmability across diverse silicone systems.

Beyond mechanical tunability, we investigated the adaptive behavior enabled by dynamic bond exchange, focusing on shape reconfiguration and self‐healing. As illustrated in Figure 2g, flat D10 strips were twisted or bent and then annealed at various temperatures for 1 h. Higher annealing temperatures enabled enhanced permanent shape reconfiguration, consistent with faster dynamic exchange. At 25 °C, no permanent shape reconfigurability was observed, which confirms that the bond exchange is inactive at room temperature. Furthermore, we observed a novel self‐healing behavior, wherein damaged samples not only recovered integrity but also exhibited enhanced stiffness from damage. In Figure S15 (Supporting Information), all self‐healed samples exhibited a notable increase in elastic modulus compared to the pristine sample, rising from 0.35 MPa (pristine) to 0.68 MPa (healed at 80 °C) and 1.33 MPa (healed at 150 °C), indicating mechanical strengthening. This behavior contrasts with typical self‐healing materials, which generally aim to restore mechanical properties to their original state. We attribute this strengthened self‐healing performance to distinctive exchange pathways in silicone networks that enable self‐healing and mechanical strengthening simultaneously. The self‐healing efficiency was calculated based on the elongation at break (ε) using the following equation: (ε_*healed*_/ε_*pristine*_)×100%. The self‐healing efficiency is 9% after healing at 80 °C and 7.4% for healed at 150 °C. The relatively low recovery is attributed to limited interfacial contact, likely hindered by the presence of silica fillers, as well as shrinkage‐induced misalignment resulting from the loss of volatile cyclic monomers and filler content during thermal treatment.

Remarkably, the dynamic behavior of our silicone material is strongly dependent on both P₄‐*^t^* Bu concentration and applied temperature. The formation and subsequent evaporation of cyclic volatile siloxanes from intra‐chain exchange provides a route for network disassembly by shifting the dynamic equilibrium toward depolymerization. When the P₄‐*^t^* Bu concentration is increased to 5 wt.% and the material is thermally annealed at 150 °C for 3 h, macroscopic disassembly of materials is observed. Importantly, this on‐demand destruction only occurs under conditions of high catalyst loading (≥5 wt.%) and elevated temperature (≥150 °C). Under these conditions, the high concentration of P₄‐*^t^* Bu undergoes nucleophilic attack on the siloxane backbone, generating silanolate intermediates that initiate both inter‐ and intra‐chain dynamic bond exchanges. Upon thermal treatment at 150 °C, the exchange reactions are accelerated, and cyclic siloxanes are rapidly volatilized and removed from the network. This continuous removal of cyclic products drives the equilibrium forward, promoting further breakdown of the network and ultimately leading to complete disassembly. In contrast, at lower P₄‐*^t^* Bu loadings (1wt.%), the dynamic network exhibits mechanical strengthening rather degradation. This is attributed to the insufficient catalyst concentration to drive extensive bond exchange and to the gradually thermal deactivation of P₄‐*^t^* Bu at 150 °C with time, limiting its catalytic activity under these conditions. Overall, we have demonstrated both adaptivity and topological programmability in commercially available polymer networks using P₄‐*^t^* Bu‐catalyzed dynamic siloxane exchange. This dual‐function mechanism enables controllable mechanical tuning, shape reconfiguring, self‐healing, and on‐demand destruction, establishing a robust and scalable platform for sustainable, reconfigurable soft robotic systems.

### 2.4 Programmable Capacitive Sensing Performance Enabled by Dynamic Silicone Dielectrics

**Figure 3:**
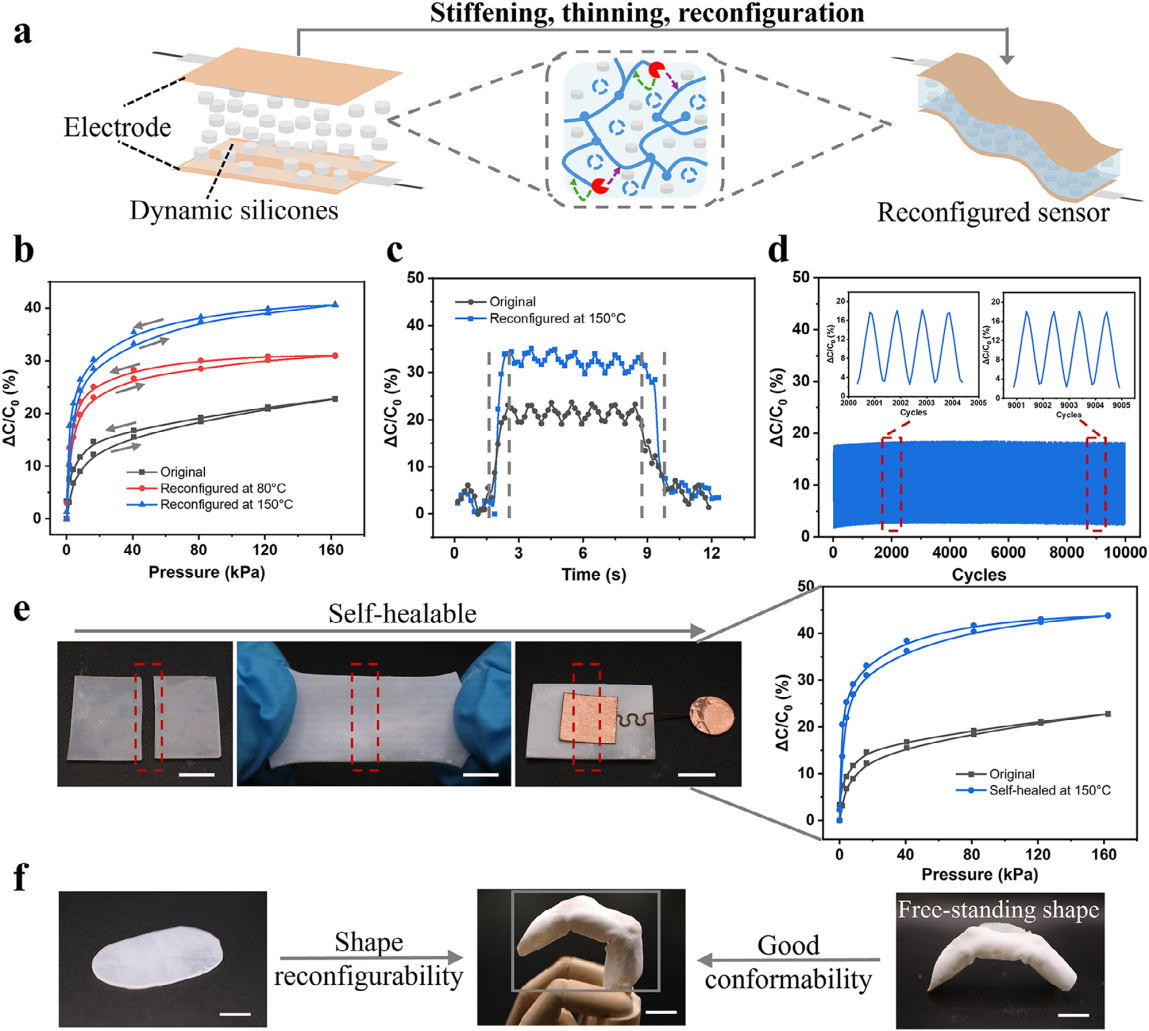
Programmable capacitive sensing performance enabled by dynamic silicone dielectrics. a) Scheme for reconfigurable capacitive sensors with the ability of stiffening, thinning and reconfiguration, utilizing dynamic silicones as the dielectric layer with inter‐ and intra‐chain siloxane bond exchange mechanisms; b) Relative capacitance change versus pressure for original and thermally reconfigured sensors; c) Response and recovery time of original and reconfigured sensors; d) Cyclic loading and unloading behaviours of reconfigured sensors over 100 cycles. The inset shows magnified views of the boxed region; e) Self‐healable capacitive sensor that recovers functionality and exhibits improved sensitivity following mechanical damage. Self‐healing proceeds at 150 °C for 3 h; All scale bars are 10 mm. f) Shape reconfiguration into finger‐like geometries for reliable and precise detection of bending motion. For shape configuration experiments, samples all thermally annealed at 150 °C for 3 h. All scale bars are 10 mm.

Notably, the ability to on‐demand program mechanical properties combined with self‐healing, shape reconfigurability and controlled destruction is promising for the omni‐adaptive robotic skin. Building on this platform, we turn our focus toward one of the primary sensing functions of human skin: pressure sensing.^[^ ^35^, ^36^ ^]^ This feature is particularly relevant for robotic skins, as it enables tactile perception for safe human interaction and precise handling of soft or delicate objects. **Figure** **3**a illustrates the schematic design of reconfigurable robotic skin integrated with capacitive pressure sensors, where dynamic silicone materials serve as the dielectric layers. As discussed previously, these materials exhibit mechanical tunability, self‐healing, shape reconfiguration, and on‐demand degradation. Here, we investigate how such material adaptivity influences capacitive sensing performance. Unless otherwise noted, all dynamic materials contain 1 wt.% P₄‐*^t^* Bu. The OmniAdapt network demonstrates a unique combination of mechanical recovery and performance enhancement: following thermal reconfiguration, the material regains structural integrity while exhibiting increased stiffness. After thermal treatment, the material maintains high deformability, with strain at break exceeding 300%, remaining above 80% after self‐healing at 80 °C, and above 50% after 150 °C. These values are well within the functional strain range for soft robotic and wearable skin applications, which typically undergo deformations of up to 30% during motion at joints. This margin ensures that the healed material retains sufficient stretchability to operate reliably as a compliant sensing interface.^[^ ^37^, ^38^ ^]^ As shown in Figure 3b, the original sensor (without thermal treatment) demonstrates a sensitivity of 1.68 kPa^−1^ under low pressure (<5 kPa). Upon thermal treatment, the sensor is reconfigured and exhibits a significantly enhanced sensitivity of 3.82 kPa⁻¹ for 80 °C and 4.67 kPa⁻¹ for 150 °C under the same pressure range, highlighting the material's potential for soft and flexible capacitive sensing applications. The thermally reconfigured sensor also demonstrates a low detection limit and a pressure resolution of 8 Pa (Figure S16, Supporting Information), enabling the detection of subtle pressure variations, particularly advantageous for robotic skin applications. To understand this improvement through thermal reconfiguration, we refer to the fundamental capacitance equation C = ε_r_ ε_0_ A/d, where two material‐dependent parameters are crucial: the relative permittivity ε_r_ and the electrode distance d. Full details of calculation are provided in the Experimental Section. Measurements under zero pressure show a slight decrease in ε_r_ from 3.17 to 3.04 after thermal reconfiguration at 150 °C. This reduction could stem from the formation of microvoids and air gaps due to the thermal evaporation of cyclic siloxane monomers (e.g., D_4_, D_5_, D_6_) and partial detachment of silica fillers. These voids and air gaps, while reducing overall permittivity, deform more easily under pressure, contributing to higher sensitivity.^[^ ^39^, ^40^ ^]^ Moreover, thermal reconfiguration resulted in a reduced dielectric layer thickness ≈ 77% shrinkage, from 1.38 mm to 1.06 mm, due to the removal of cyclic volatile monomers and fillers. This thickness reduction increases the baseline capacitance and amplifies relative changes under applied pressure, further improving sensitivity.^[^ ^41^ ^]^ These results highlight how mechanically tunable silicones through dynamic bonds exchange can be leveraged to enhance sensor performance toward higher sensitivity. Besides, all sensors exhibit minimal hysteresis during loading and unloading. One big challenge associated with dynamic covalent polymers for use in robotic applications is their inherent viscoelasticity due to dynamic bond exchange,^[^ ^42^ ^]^ which can lead to signal drift. It is worth noting that, in our system, the dynamic siloxane exchange reactions are inactive at room temperature, leading to elastic rather than viscous plastic deformation. Consequently, minimal hysteresis in sensing is observed. Response and recovery times were also evaluated (Figure 3c), revealing a faster response time of 0.468s in the reconfigured sensors, compared to 0.78s in the original. Recovery times are similarly improved: 0.624 s for the reconfigured versus 0.936 s for the original. These enhancements arise from the thermally induced structural changes in the dielectric film. Specifically, shrinkage and partial loss of cyclic siloxanes results in a thinner dielectric layer and reduce the stress propagation path. This minimizes viscoelastic damping, enabling faster mechanical relaxation and quicker signal transmission, making the reconfigured sensor well‐suited for real‐time sensing applications. Long‐term performance stability was demonstrated through cyclic pressure tests, with the reconfigured sensor maintaining stable output over 10000 cycles in Figure 3d, showing excellent repeatability, reliability and underlining the sensing robustness of the material. Additional data for the pristine sensor (Figure S17, Supporting Information) confirm similar stability for over 5800 cycles, reinforcing that the siloxane exchange remains inactive at ambient temperature. The overall performance of the OmniAdapt sensor is compared to other state‐of‐the‐art devices in Table S1 (Supporting Information), highlighting the good performance of our sensor, underscoring its relevance and competitiveness within its field.

**Figure 4:**
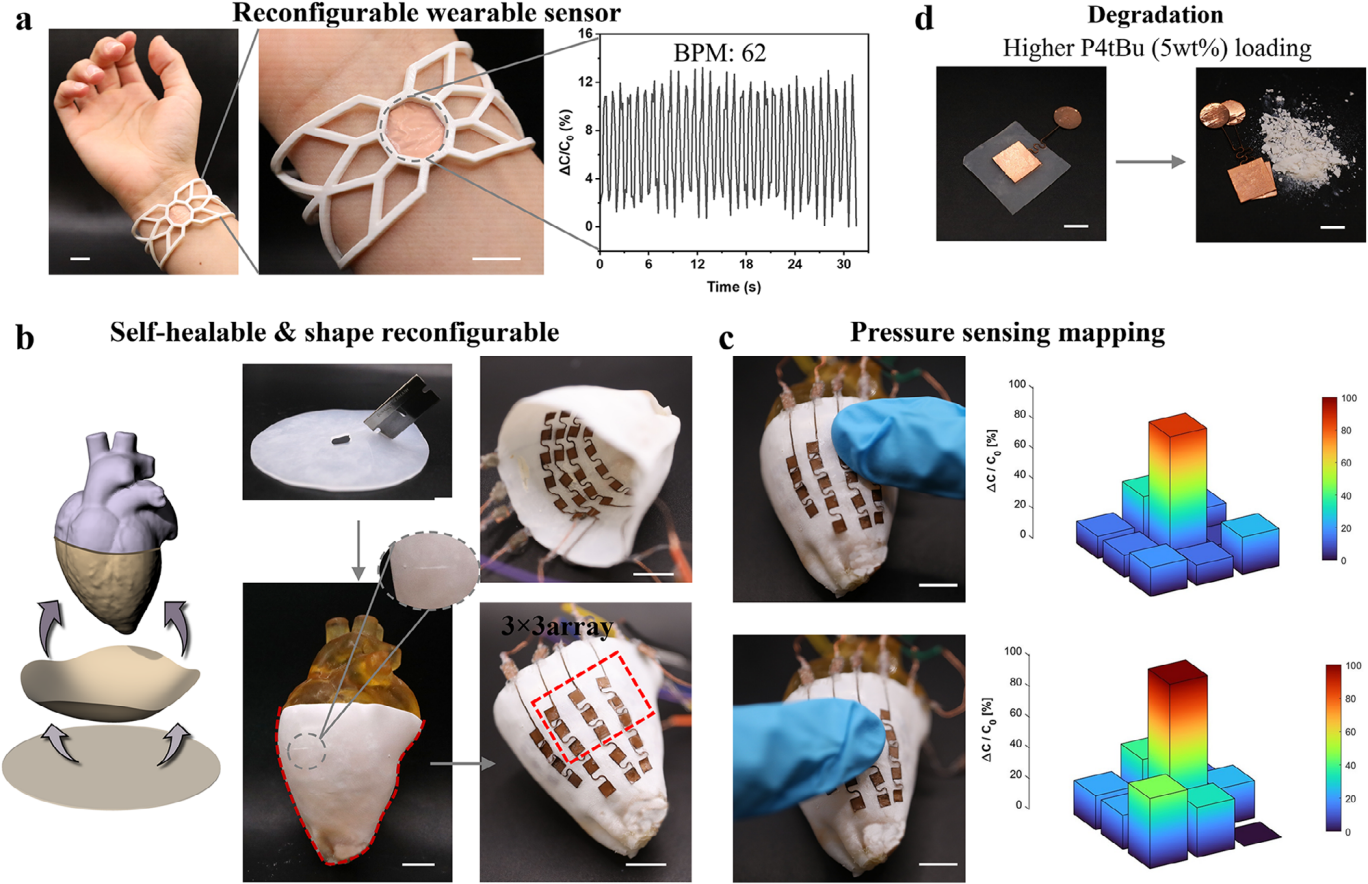
Omni‐adaptive robotic skins. a) Reconfigured sensor integrated into a 3D‐printed wearable device for real‐time pulse monitoring; b) Self‐healable and shape‐reconfigurable robotic skin using an embedded electrode array; Enlarged picture shows self‐healing from damage; c) Pressure sensing mapping within the self‐healable and shape‐reconfigurable robotic skin (*x*‐ and *y*‐axis: position of applied pressure, *z*‐axis: relative capacitance change); d) On‐demand degradable sensors with higher P₄‐*^t^* Bu (5 wt.%) loading after thermal treatment at 150 °C for 3 h. All scale bars are 10 mm.

In addition to mechanical tuning, the sensor also exhibits unique self‐healing behavior that not only restores functionality, but also enhances sensitivity from damage. This remarkable phenomenon is attributed to the dynamic silicone dielectrics that enable the distinctive inter‐chain and intra‐chain exchange pathways. Specifically, the damaged interfaces are healed through the reformation of siloxane via bonds exchange reactions in Figure 3e, rendering damage sites indistinguishable. Concurrently, intra‐chain exchanges induce the isomerization of silicone network, resulting in a reconfigured structure with better sensing sensitivity compared to the pristine state. This appealing self‐healing is distinct from conventional strategies aimed merely at restoring and recovering pre‐damage performance. Instead, our method offers the potential for functional improvement from damage, which could be significant interest for intelligent, sustainable robotics capable of adaptive repair. Besides, shape reconfigurability was explored. As illustrated in Figure 3f, a planar film can be permanently reconfigured into complex geometries, such as finger‐like structures, enabling excellent conformability to irregular surfaces like a robotic hand. This facilitates precise and reliable motion sensing. Unlike previous designs that rely on external stress to maintain structural shape, our reconfigurable materials are freestanding and structurally stable, ensuring consistent sensor‐substrate contact reliable sensing. It is important to note that the dynamic behavior of these materials is temperature‐dependent. The catalytic activity of P₄‐*^t^* Bu diminishes after prolonged exposure (3 h) above 150 °C, due to thermal deactivation, which could limit multiple times programming. Nevertheless, the integrated functionalities of mechanical programmability, self‐healing, and permanent shape reconfigurability make dynamic silicone materials a promising platform for the OmniAdapt. These properties are particularly advantageous for applications such as robotic skin, which is designed to replicate the mechanical compliance and sensory capabilities of human skin, requiring softness, flexibility, stretchability, and the ability to conform to irregular surfaces. The OmniAdapt dielectric layer fulfills these criteria, enabling its integration into capacitive sensing arrays with high sensitivity and spatial resolution (**Figure** **4**c).

### 2.5 On‐Demand Reconfigurable, Self‐Healable and Degradable Robotic Skins Built with Pressure Capacitive Sensor

Building upon the adaptive sensing performance of capacitive sensors by employing dynamic silicones as dielectric layers, we further explored their applicability in the omni‐adaptive robotic skin. In Figure 4a, the OmniAdapt material enables structurally reconfigured capacitive sensors with enhanced sensitivity, allowing it to be adapted into a wearable device for physiological monitoring. We demonstrate a pulse sensor integrated into a 3D‐printed bracelet to capture arterial pulse signals with high fidelity, yielding a measured heart rate of 62 beats per minute (BPM). The enhanced sensitivity of the sensor stems from the network structure changes enabled by dynamic siloxane bonds, which tune the material's thickness and stiffness to amplify the pressure‐induced capacitance response. Precise control of physiological markers is critical for real‐time health monitoring, improving personalized medicine and enabling early disease detection. We note that the programmability of our sensor makes it a good candidate for precise recordings, as each sensor can be tuned for optimal performance depending on the target placement and target signal. Sensor array with individually tuned sensitivities could simultaneously capture diverse physiological signals such as pulse, blood pressure or respiration, enabling optimal health tracking.

Beyond wearable applications, the dynamic material exhibits excellent potential for integration into robotic skin systems with shape‐reconfigurable and self‐healing capabilities, two features that can be particularly useful for haptic feedback platforms in surgical robotics. Haptic feedback is essential for both training and performing minimally invasive surgeries,^[^ ^43^ ^]^ where the absence of tactile sensation can compromise precision and increase the risk of damaging the tissue. It enables surgeons to differentiate between tissue types, control insertion force and navigate in complex anatomical environments to avoid surgical errors and improve accuracy. In the demonstration shown in Figure 4b, an initially planar film subjected to mechanical damage via a blade incision, was thermally annealed at 150 °C for 3 h. This treatment triggered dynamic intra‐ and inter‐chain siloxane exchange reactions inherent to the P₄‐*^t^* Bu‐based silicone network, enabling simultaneous self‐healing and permanent shape reconfiguration into a 3D, heart‐shaped geometry. The reshaped dielectric film has an excellent conformability to complex surfaces, such as concave and convex curvatures of the heart‐shaped mold (indicated by the red dashed outlines), which is essential for in situ integration onto arbitrary shaped biological tissue during robotic procedures. The self‐healing property of the dielectric film further allows the sensor to fully recover its functionality, after a local puncture or tear. The enlarged picture highlights the fully healed region, confirming the material's intrinsic self‐repairing capability. To assess the integrated sensor functionality in a reshaped configuration, 3  ×  3 electrode arrays (outlined in red dashed lines) were aligned onto the inner and outer side of the self‐healed, heart‐shaped robotic skin, to perform spatial pressure sensing. The array has good real‐time response due to robust electrical connectivity. The resulting pressure mapping, presented in Figure 4c, demonstrates precise localization of applied pressure stimuli across the surface, confirming the spatial resolution and responsiveness of the self‐healed and shape‐reconfigured robotic skin. In detail, directional pressure from the right side selectively activated the corresponding electrode regions, indicating localized sensitivity and spatial resolution. Notably, these on‐demand adaptive functionalities (shape and mechanical reconfiguration, self‐healing) are attributed to intrinsic dynamic silicone networks, which offer distinctive inter‐chain and intra‐chain siloxane exchange reactions within P₄‐*^t^* Bu.

Furthermore, we investigated the material's potential for controlled degradation, a critical feature for robotic applications.^[^ ^44^, ^45^ ^]^ Such functionality is increasingly important in contexts where self‐destruction is required to ensure data security following mission completion, or in sustainable electronics aimed at minimizing plastic waste. To achieve this, we increase the P₄‐*^t^* Bu concentration to 5 wt.% and observed enhanced dynamic exchange reactions upon thermal treatment (150 °C, 3 h), which facilitated the generation and release of volatile cyclic siloxanes. This process led to a significant mass loss of ≈70% before and after thermal annealing, indicating substantial structural disassembly and demonstrating the feasibility of on‐demand, thermally programmed material degradation (Figure 4d). Overall, these findings underscore the programmable and dynamic characteristic of our commercially available silicones. The OmniAdapt built with pressure capacitive sensor not only exhibits mechanical and functional reconfigurability, autonomous healing, but also offers a route toward on‐demand degradation. These appealing attributes render dynamic silicones as a promising material platform for next‐generation soft robotic systems with intelligent, adaptive, and sustainable performance.

## 3 Conclusion

In summary, we present an omni‐adaptive robotic skin that integrates shape adaptivity, performance programmability, self‐healing and on‐demand destructibility within a single robotic material. This multifunctionality is enabled by commercially available dynamic silicone materials that can undergo two distinctive siloxane bond exchange pathways. Upon incorporation of a superbase phosphazene catalyst, silanolate species are generated, which facilitate nucleophilic attack on siloxane bonds through either intra‐chain or inter‐chain exchange routes. These exchanges endow shape reconfigurability and self‐healing. Remarkably, intra‐chain exchanges result in the formation of volatile cyclic siloxanes that evaporate from the network, which causes the loss of silica fillers. We note that the weight loss of cyclic monomers is significantly higher than that of the silica fillers. This selective mass loss leads to a relative enrichment of the silica content, which contributes directly to mechanical reinforcement. It allows for polymer network isomerization and correspondingly mechanical programmability. This behavior distinguishes our dynamic siloxane network from conventional dynamic covalent polymer systems, in which the overall network architecture remains unchanged during bond exchange, thus lacking mechanical programmability.

Leveraging this dynamic material as the dielectric layer for capacitive pressure sensor, the omni‐adaptive robotic skin was developed. Programmable and reconfigurable sensing performance were achieved due to the distinctive exchange pathways in the dynamic silicones. Importantly, a novel self‐healing sensing behavior was observed, wherein damaged sensors not only recovered functionality but exhibited enhanced performance, characterized by increased sensitivity. Furthermore, the skin exhibits shape reconfigurability to a heart model, revealing its potential for haptic feedback in minimally invasive surgery. By driving the dynamic equilibrium toward the formation of cyclic siloxanes under higher P₄‐*^t^* Bu loadings, the skin undergoes on‐demand destruction. Together, these capabilities highlight promising applications from wearable devices, haptic feedback for MIS practice to self‐healing and on‐demand destructible robotic skin.

## 4 Experimental Section

### Materials

Dragon Skin 10, 30, and Ecoflex 50 were purchased from Kaupo. Phosphazene base P₄‐*^t^* Bu solution and Poly(dimethylsiloxane), vinyl terminated (Mw∼25000) was obtained from Sigma‐Aldrich. Toluene was purchased from Thermo Scientific. Copper coated PI was obtained from KONLIDA. All chemicals were used without further purification.

### Model Experiment

Poly(dimethylsiloxane) (1 g) was mixed with an equal mass of toluene. P₄‐*^t^* Bu was added in varying concentrations ranging from 0.25 wt.% to 1 wt.% relative to the weight of poly(dimethylsiloxane). The resulting mixture was magnetically stirred at 25 °C for 3 h. For temperature‐controlled experiments, the mixture was stirred under identical conditions at either 60 °C or 90 °C for 3 h.

### Reconfigurable Capacitive Sensor Fabrication

The dielectric layer was prepared using Dragon Skin 10. For the preparation of Dragon Skin 10, Part A and Part B were mixed in a 1:1 ratio using a planetary centrifugal mixer at 2000 rpm for 120 s. The resulting mixture was spin‐coated onto a glass wafer and cured at 60 °C overnight. The thickness of the dielectric layer was controlled by adjusting the spin speed and duration.

### Post‐Swelling to Introduce the Catalyst

P₄‐*^t^* Bu was introduced via swelling in toluene solutions. Specifically, toluene was used at a volume ten times the weight of the dielectric layer, with P₄‐*^t^* Bu concentrations ranging from 0.25 wt.% to 1 wt.% relative to the dielectric mass. The dielectric films were immersed in the solution at room temperature for 1 h, then dried in a fume hood at room temperature until all residual toluene had evaporated.

Copper‐coated polyimide sheets were patterned into electrodes (2.8 cm in diameter) using an ultraviolet laser (Protolaser U4, LPKF) for characterization. The electrodes were connected to copper wires for connection using solder paste. The top side electrodes where laminated onto one side of a dielectric film, encapsulated with spin‐coated Dragon Skin 10 (2,000 rpm, 120 s) for protection, and cured at room temperature for 75 min. The process was repeated on the opposite side, aligning the capacitive electrodes.

### Shape Reconfiguration

A preswollen (as described previously) film was wrapped around a 3D printed (Stratasys PolyJet) mold and fixed with a thin polyamide net. The film was first heated to 150 °C for 3 h and then detached from the mold resulting in a freestanding structure.

### Degradation Experiment

A higher concentration of P₄‐^t^Bu (5 wt.%) was introduced into the material via swelling in a toluene solution, following the same post‐swelling procedure used for catalyst incorporation. For thermal treatment, the samples were annealed at 150 °C for 3 h.

### Mechanical Characterization

All mechanical tests were conducted with a Zwick machine under 100 mm min^−1^ tensile speed. A minimum of three standard dumbbell‐shaped specimens were tested for each sample. ^29^Si‐NMR was measured with Bruker (300 MHz) using CDCl_3_ as the solvent. Thermogravimetric analysis (TGA) was conducted with the TA Instruments. The sample was heated to 800 °C at a heating rate of 30 °C min^−1^ under air atmosphere and the weight percentage was recorded. Fourier transform infrared (FTIR) analysis was conducted by BRUKER OPUS. Energy‐dispersive X‐ray spectroscopy (EDS) was conducted using GeminiSEM 450.

### Capacitance Characterization

The capacitance response of the sensor under applied pressure was measured using four‐ point measurement with a LCR meter (LCR meter IM3533, Hioki) at a frequency of 100 Hz and 5 V applied source voltage. A force ranging from 0N to 100N (0 kPa to 160 kPa) was applied uniformly to the sensor using a test stand (Manual test stand TVL series, Sauter) and measured with a force gauge (F100 EXT digital force gauge, Sauter). Pressure was determined by dividing the applied force by the sensor's contact area. The cycle tests were conducted by applying 7 kPa onto the Omni‐Adapt sensor for 10 000 cycles with a texture analyzer (TA.XTplusC, UK), and measuring the resulting capacitance with an LCR meter (LCR meter IM3533, Hioki).

### Relative Permittivity

The relative permittivity (ε_r_) of the material was determined by measuring the capacitance between two parallel‐plate electrodes separated by 1 mm, either with air or with a 1 mm‐thick dielectric film inserted between them, minimizing systematic errors by maintaining identical geometry across measurements.

Capacitance measurements were performed using the same setup described earlier. The relative permittivity ε_r_ of the film was then calculated as:

(1) $$\varepsilon_{r}=\frac{C_{\;film}}{C_{air}}$$

where *C*_*film*_ and *C*_*air*_ are the measured capacitances with the dielectric film and with air, respectively.

Based on the parallel‐plate capacitor mode

(2) $$C=\frac{\varepsilon_{0}\varepsilon_{r}A}{d}$$

with ε_0_ =  8.85 × 10^−12^ F m^−1^ the permittivity of free space, *A* the overlapping area of the electrodes, and *d* the separation distance (equal to the film thickness). The permittivity of air was approximated as ε_*air*_ ≈ 1.

### Robotic Skin

A preswollen film (10 cm in diameter) was shape‐reconfigured around a 3D‐printed heart model as previously described. An electrode array (9 mm^2^ per electrode, with 3 mm spacing), fabricated following established protocols, was fixed to the inner and outer surfaces of the resulting freestanding structure using silicone adhesive. The pressure of a finger pressing on the sensing map was recorded as previously described and visualized through a 3D array spatial mapping implemented in MATLAB.

## Conflict of Interest

The authors declare no conflict of interest.

## Author Contributions

W.M. and L.L. contributed equally to this work. W.M. conceived and designed the research. W.M., L.L. and L.H. performed experiments and analyzed data. All authors contributed to discussion and interpretation of results. H.B. supervised the project. W.M., L.L. and H.B. wrote the paper.

## Supporting information



Supporting Information

## Data Availability

The data that support the findings of this study are available from the corresponding author upon reasonable request.
