# Supplementary material for: A Shape‐Adaptive, Performance‐Programmable, Self‐Healable and On‐Demand Destructible Robotic Skin via Self‐Strengthening Dynamic Silicone
Source: Adv Sci (Weinh). 2025 Jul 26;13(15):e08823. doi: 10.1002/advs.202508823 (PMC13042974; doi:10.1002/advs.202508823)
Supplement: Supplementary file 1 — Supporting Information [file ADVS-13-e08823-s001.docx]

**Supporting information**

**A Shape-Adaptive, Performance-Programmable, Self-Healable and On-Demand Destructible Robotic Skin via Self-Strengthening Dynamic Silicone**

Wusha Miao^1#^, Lara S. Laamari^1#^, Jing Yu^1,2^, Sanjay Schreiber^1^, Lukas Heer^1^, Jiacheng Cui^1,3^, Jiayuan Huang^1^, Hedan Bai^1^*

^1^Laboratory of Robotic Materials, Department of Materials, ETH Zürich, Hönggerbergring 64, 8093 Zürich, Switzerland

^2^Key Laboratory of Silkworm and Bee Resource Utilization and Innovation of Zhejiang Province, Institute of Applied Bioresource Research, College of Animal Science, Zhejiang University, Hangzhou, 310058, P.R. China

^3^No.2 Linggong Road, Ganjingzi District, Dalian City, Liaoning Province, 116024, P.R. China

Email: hedan.bai@mat.eth.ch


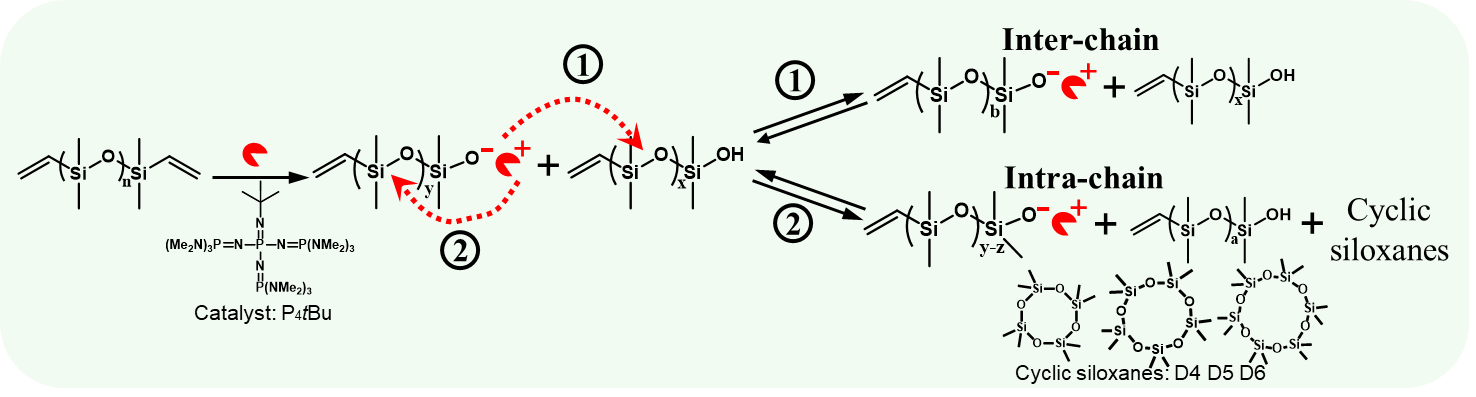


Figure S1. Molecular study of the P₄-*^t^*Bu-triggered silonate inter-chain (pathway 1) and intra-chain (pathway 2) exchange.


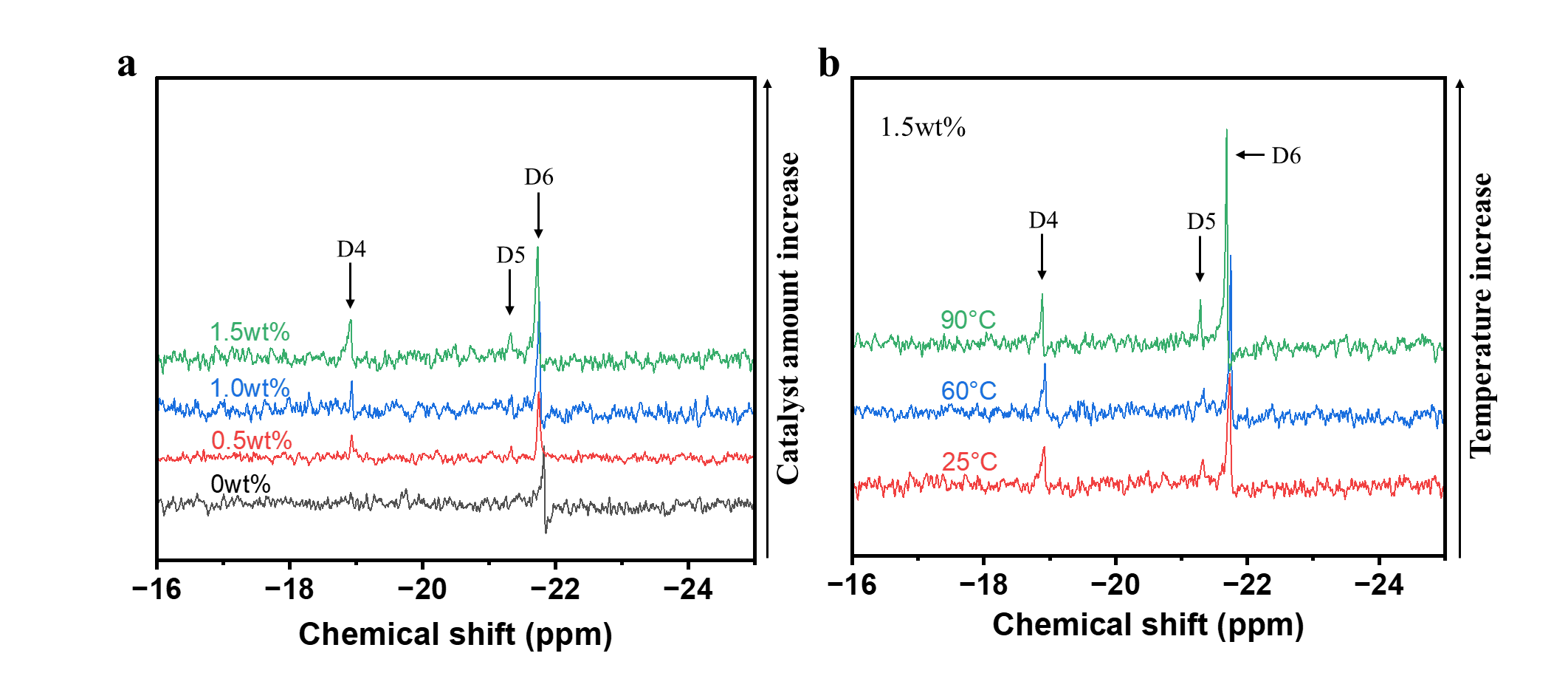


Figure S2. ^29^Si-NMR spectra of the model compounds. a) with different P₄-*^t^*Bu loading at room temperature; b) with 1wt% P₄-*^t^*Bu under different temperature annealing.


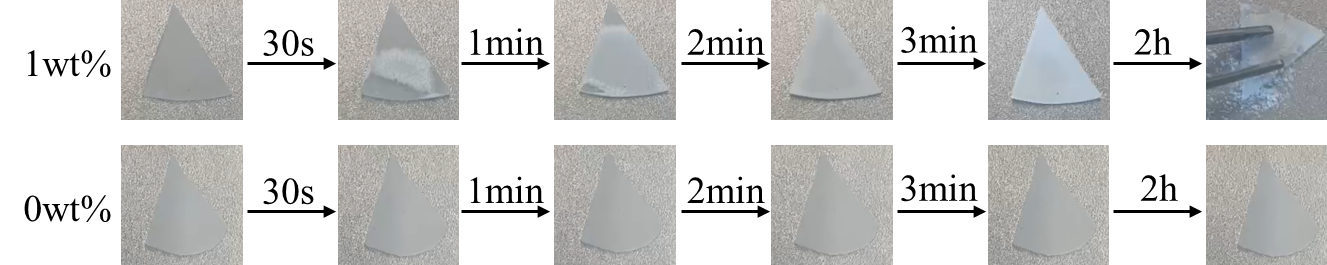


Figure S3. Macroscopic change in D10 samples with and without P₄-*^t^*Bu under 150°C.


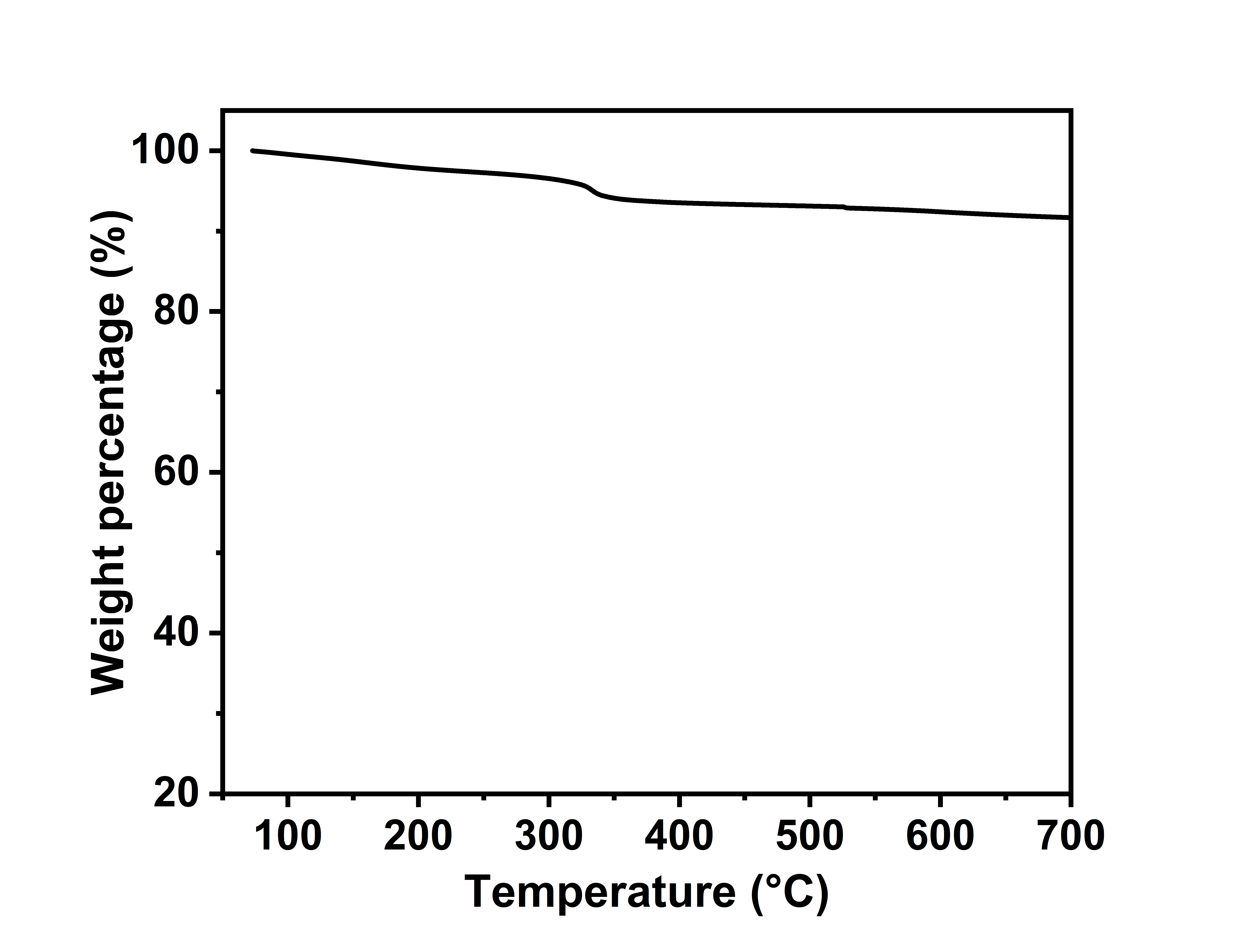


Figure S4. TGA curve of the white powder detached from D10.


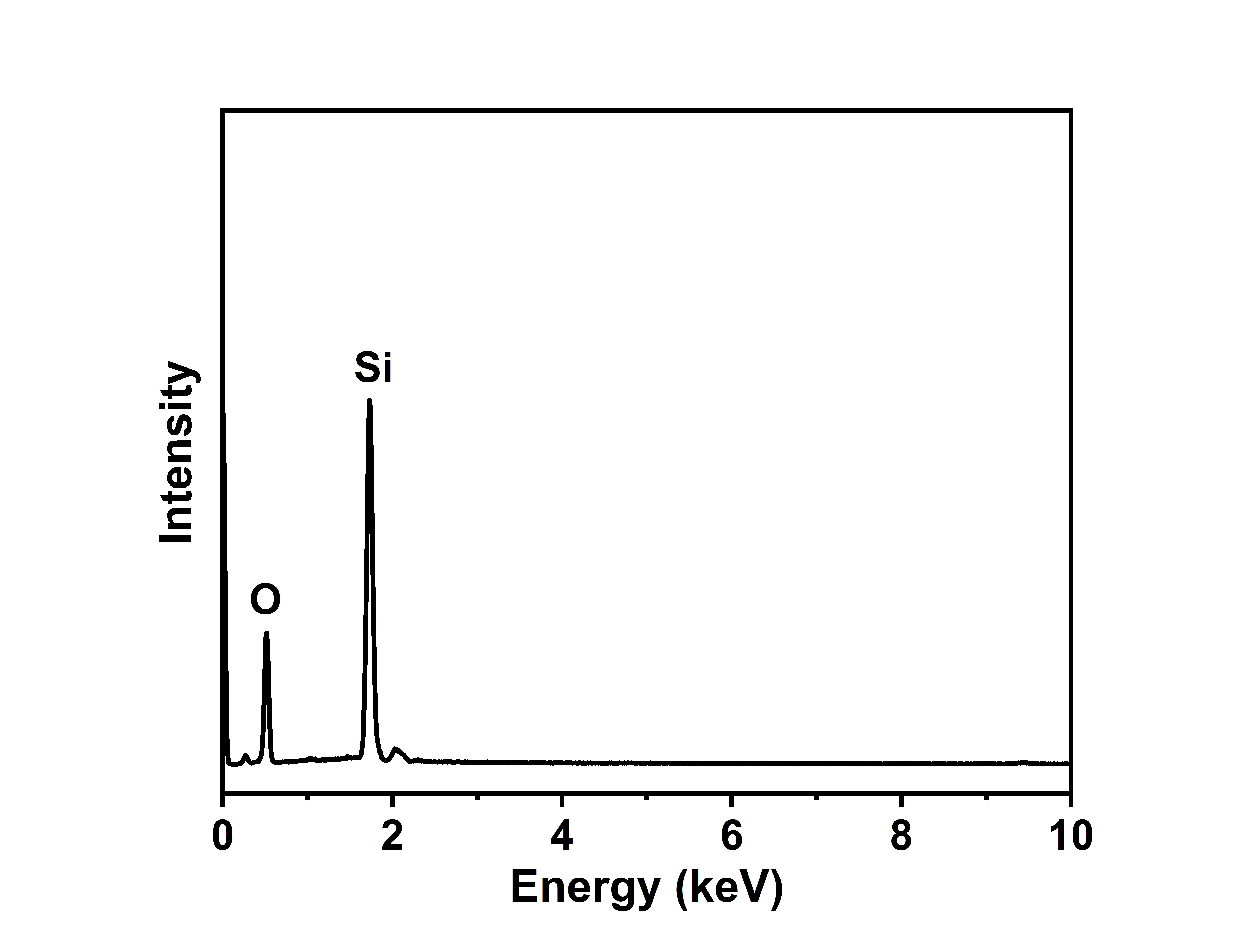


Figure S5. EDX result of the white powder detached from D10.


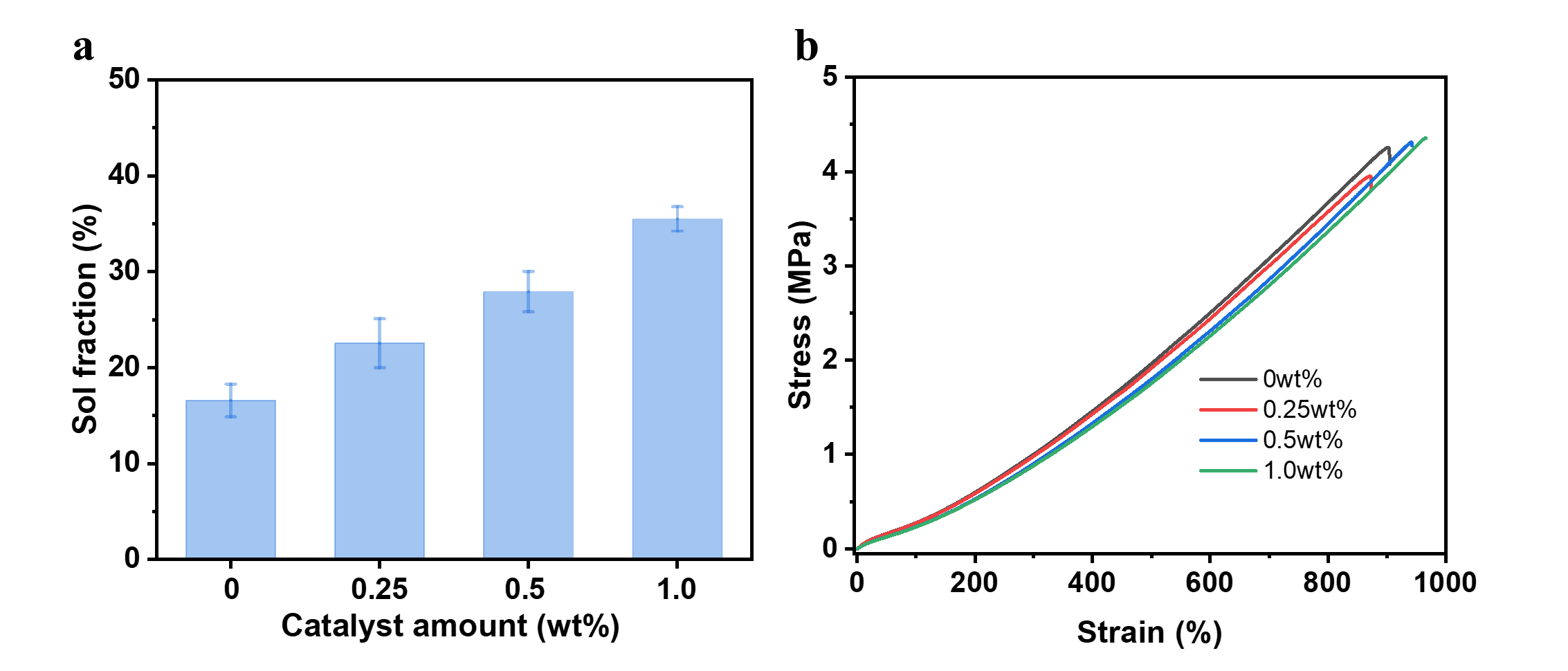


Figure S6 a) The weight loss with varying catalyst loadings before and after the post-swelling process; b) Stress-strain curves of D10 samples after the post-swelling process at room temperature.


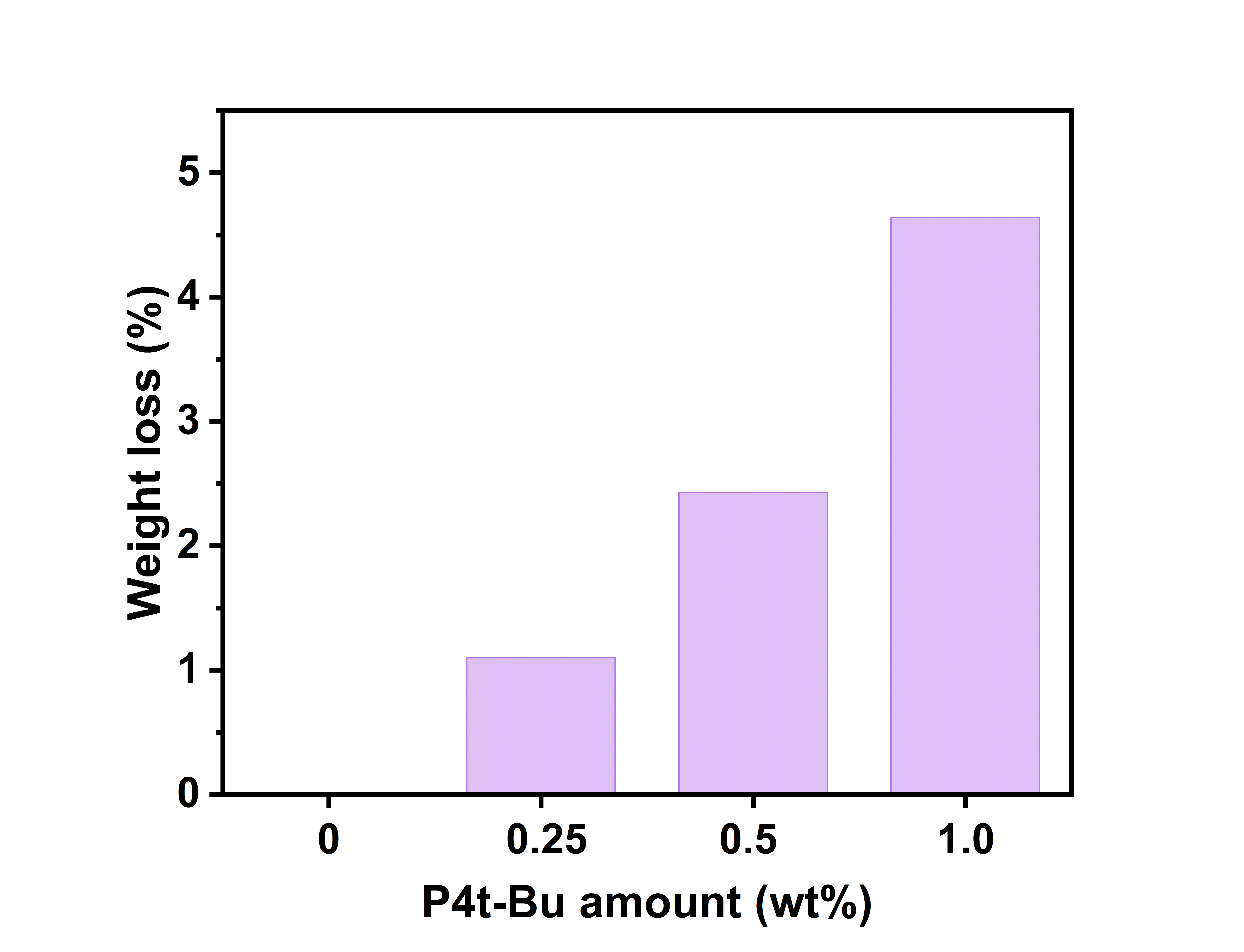


Figure S7. Weight percentage of silica fillers detached from D10 samples under different amounts of P₄-*^t^*Bu at 150°C.


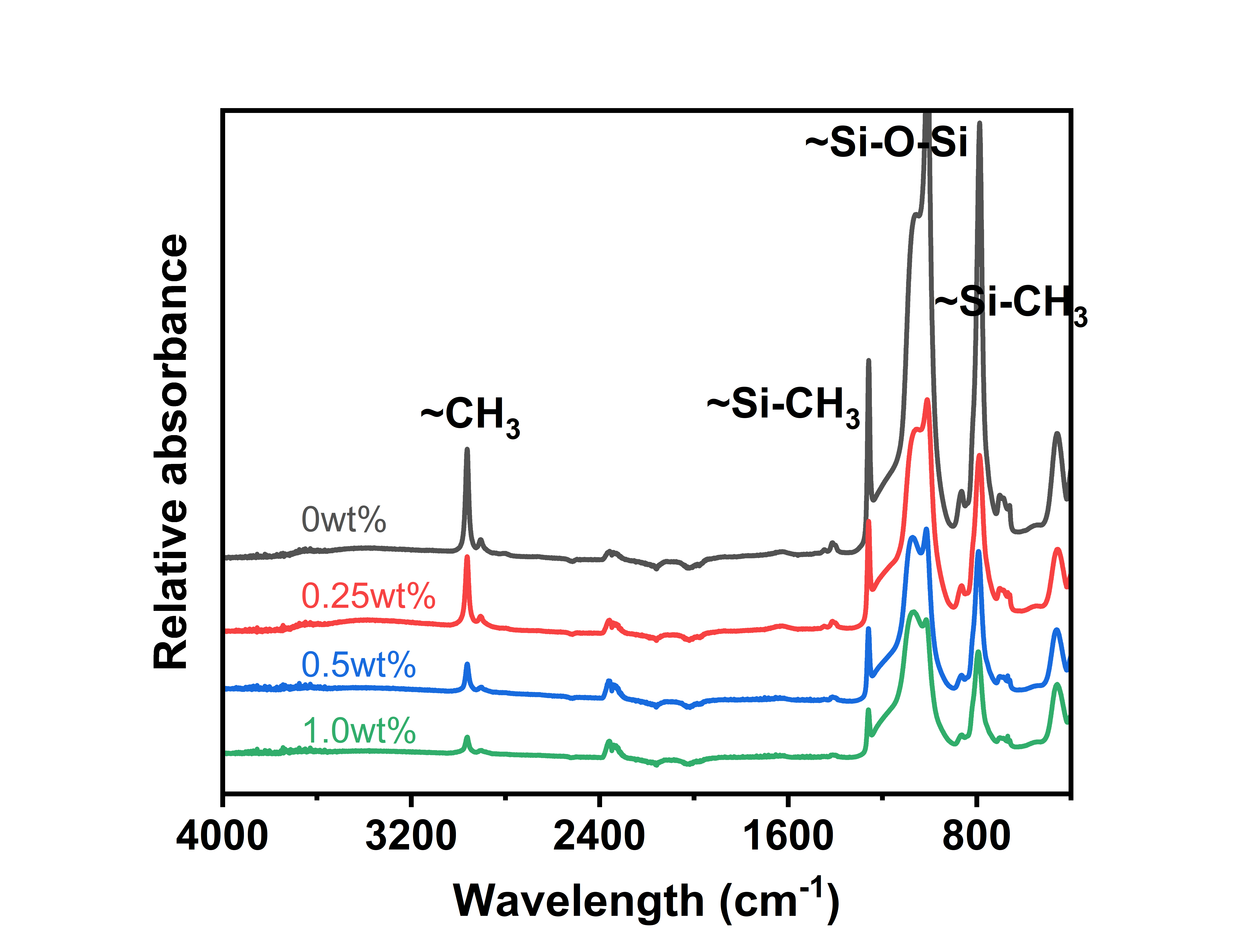


Figure S8. FTIR spectra of D10 samples with different P₄-*^t^*Bu loading.


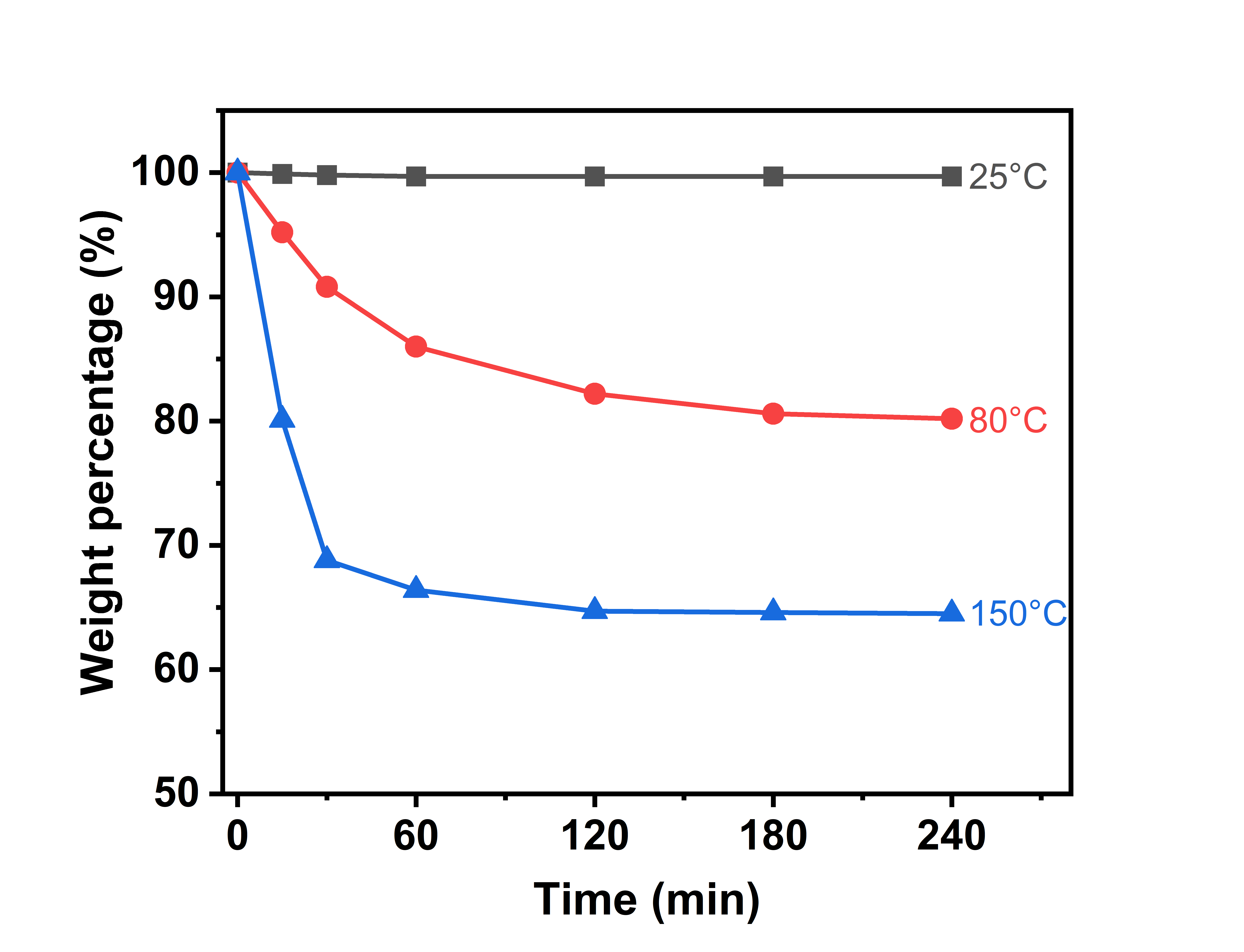


Figure S9. Weight change from cyclic monomers (D_4_, D_5_ and D_6_) with time under different temperature within 1wt% P₄-*^t^*Bu for D10.


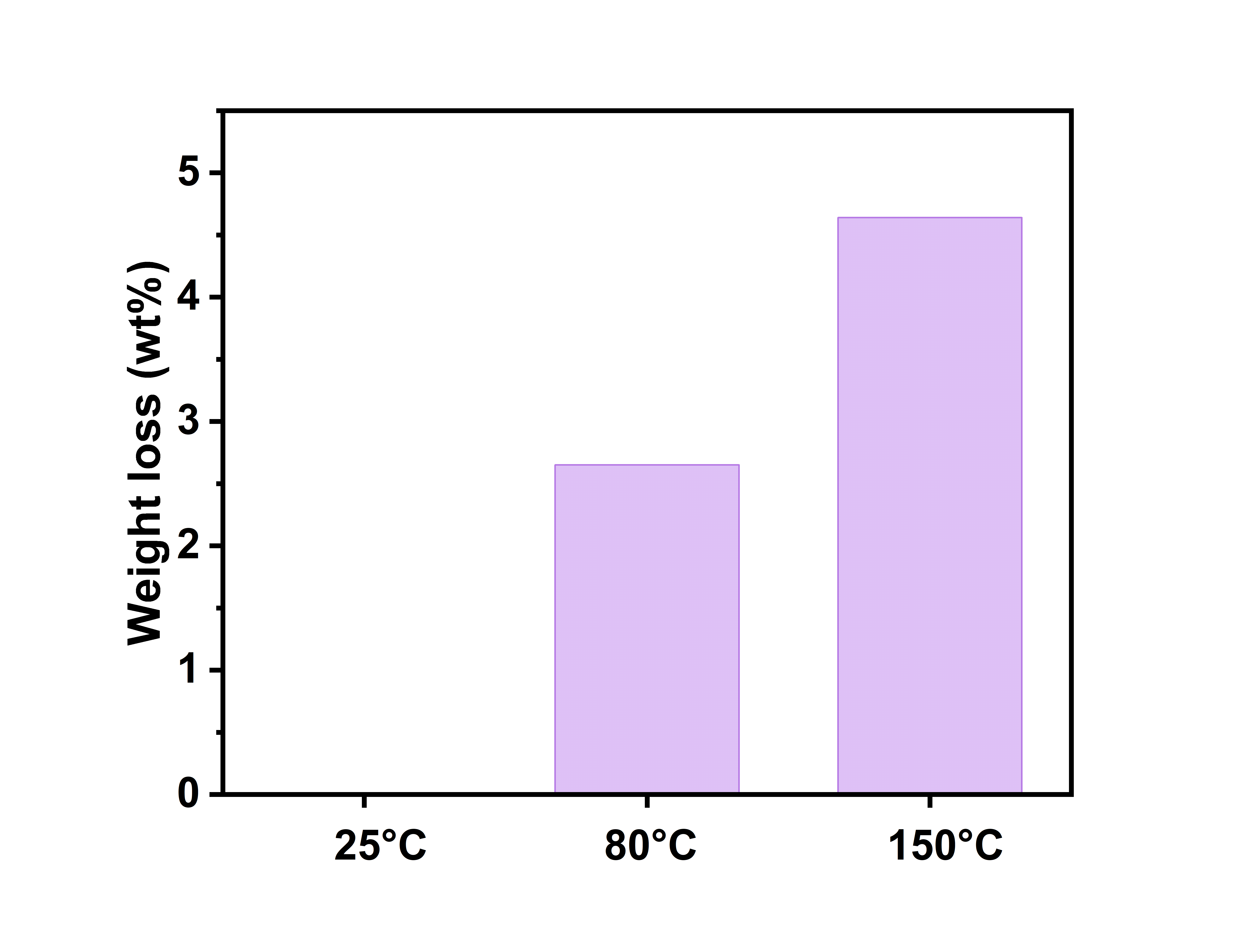


Figure S10. Weight percentage of silica fillers detached from D10 samples under different temperature within 1wt% P₄-*^t^*Bu.


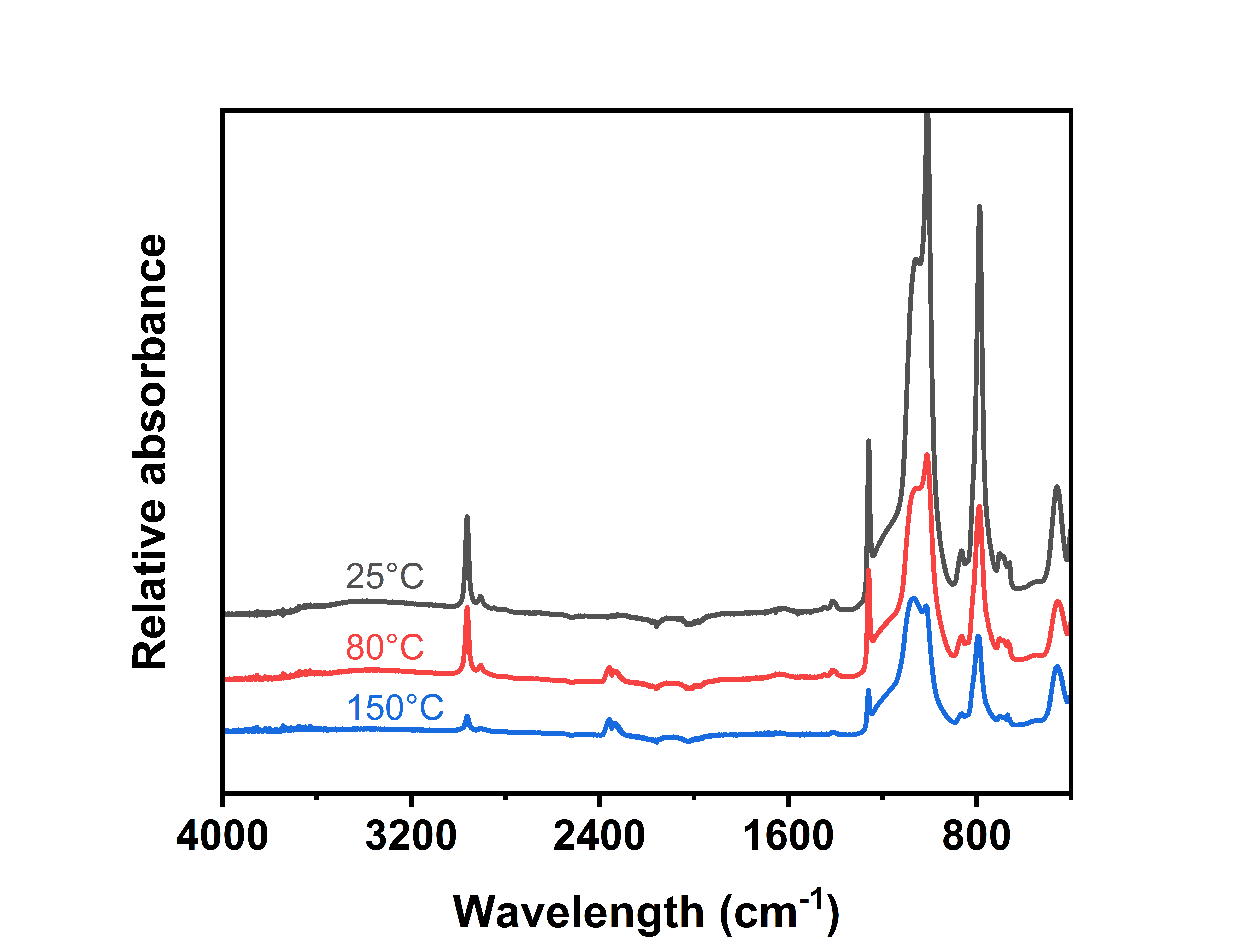


Figure S11. FTIR spectra of D10 samples under different temperature within 1wt% P₄-*^t^*Bu.


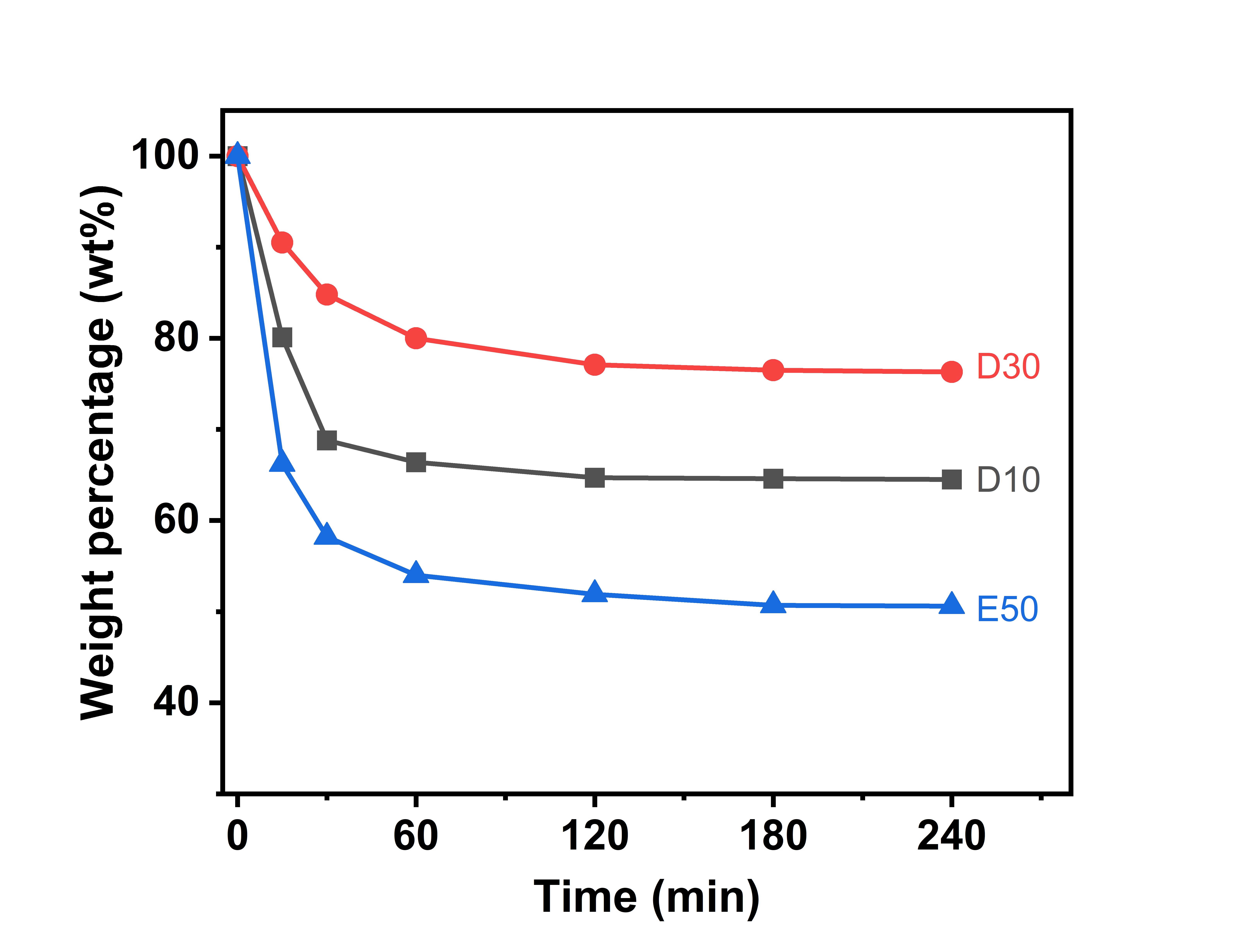


Figure S12. Weight change from cyclic monomers (D_4_, D_5_ and D_6_) for different silicones at 150°C.


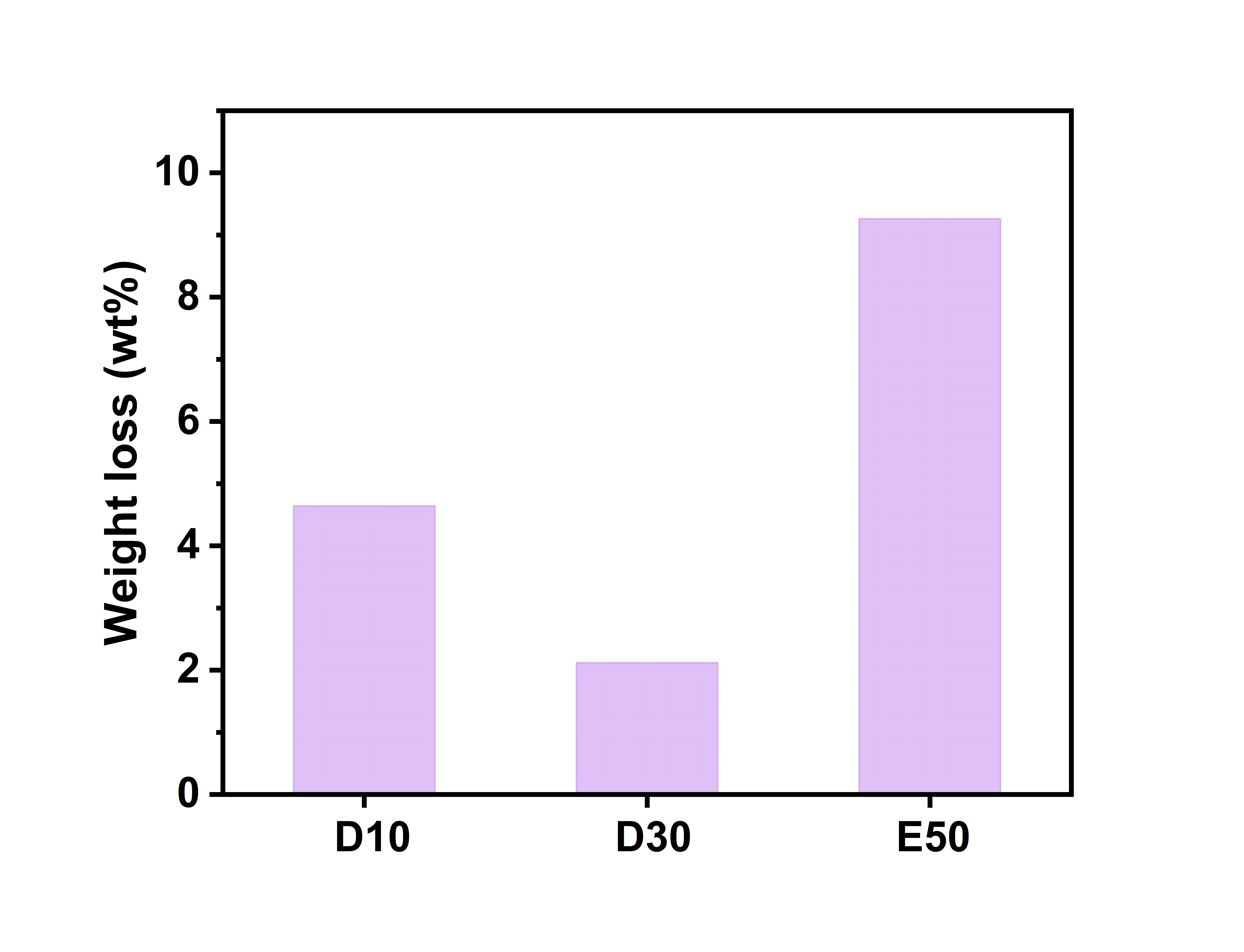


Figure S13. Weight percentage of silica fillers detached from different silicone silicones at 150°C.


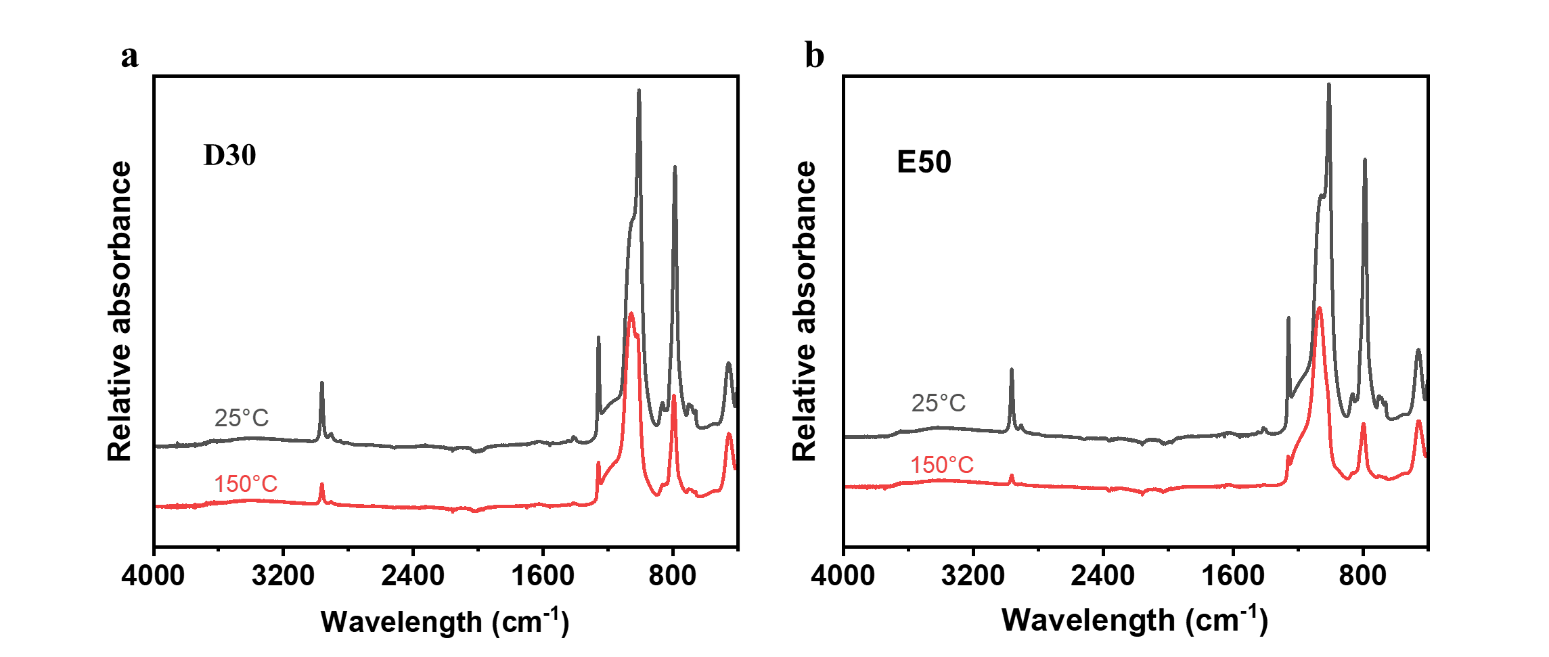


Figure S14. (a) FTIR spectra of D30 samples before and after thermal treatment at 150°C within 1wt% P₄-*^t^*Bu; (b) FTIR spectra of D30 samples before and after thermal treatment at 150°C within 1wt% P₄-*^t^*Bu.


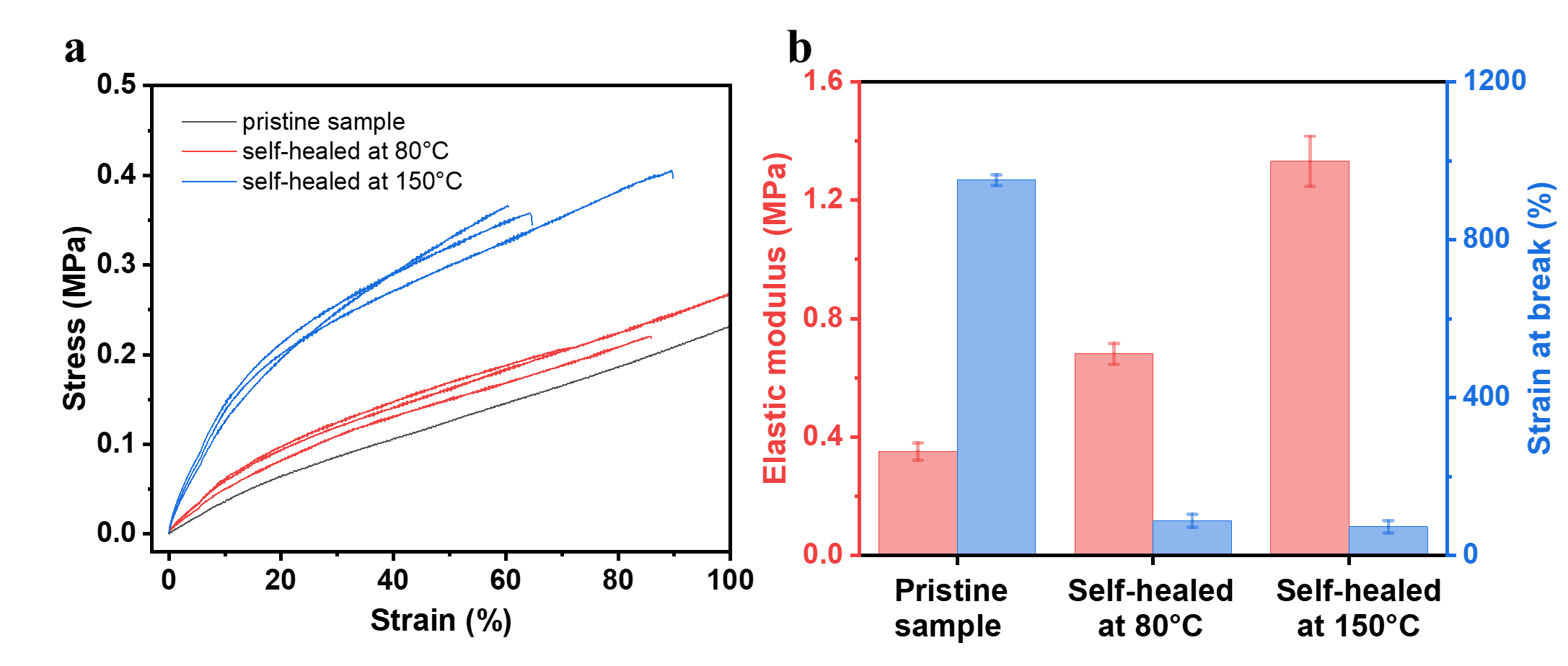


Figure S15. Self-healing performance of mechanical properties. (a) Stress-strain curves of self-healed D10 samples containing 1 wt% P₄-tBu; (b) Comparison of elastic modulus and strain at break between pristine and self-healed D10 samples.


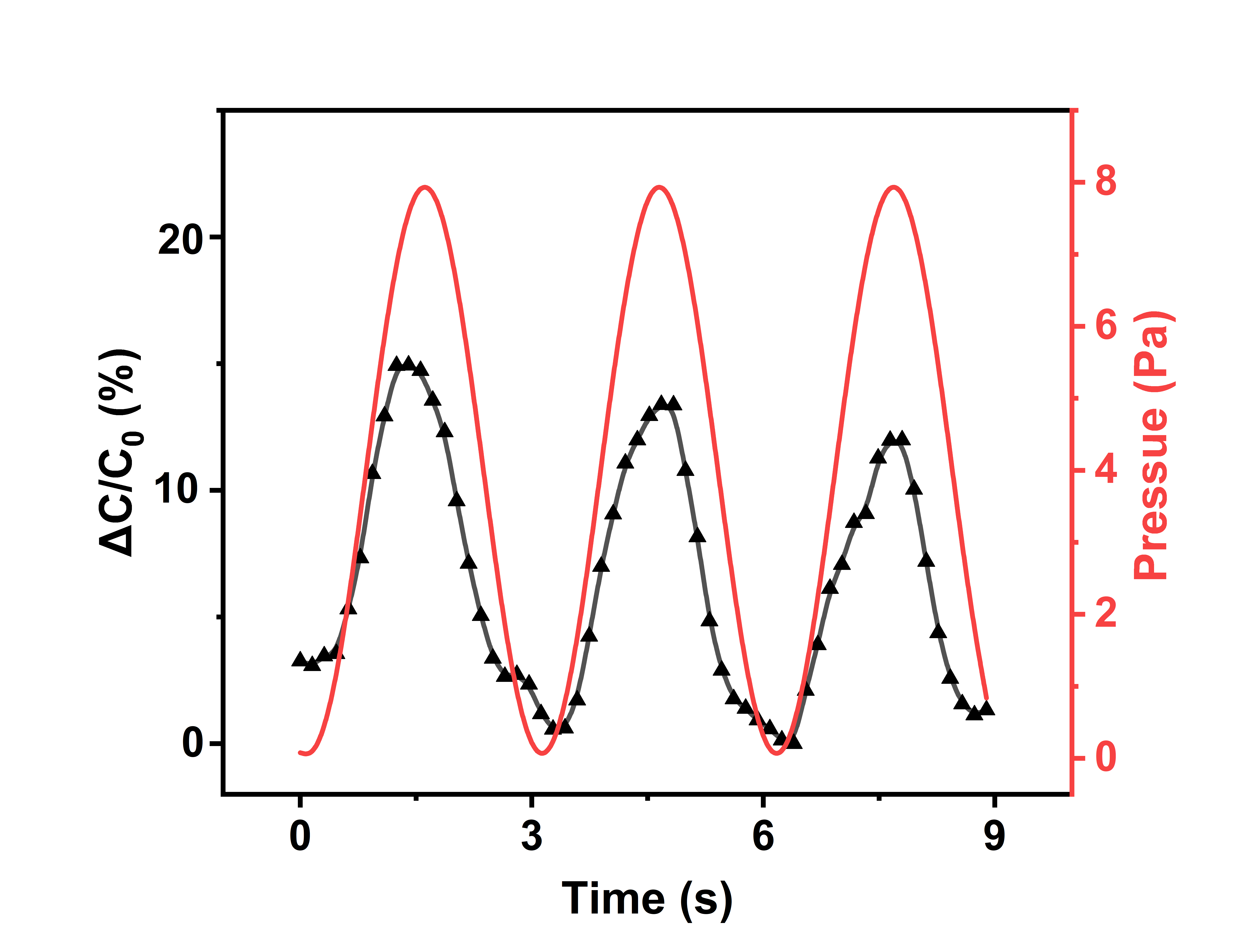


Figure S16. Relative capacitance change over time for thermally reconfigured sensors under repeated application of 8 Pa pressure. The resolution of the measurement setup is the limiting factor. Pressure increments below 8 Pa result in a signal-to-noise ratio that is too low for reliable detection. Consequently, the effective pressure resolution and limit of detection is limited to 8 Pa.


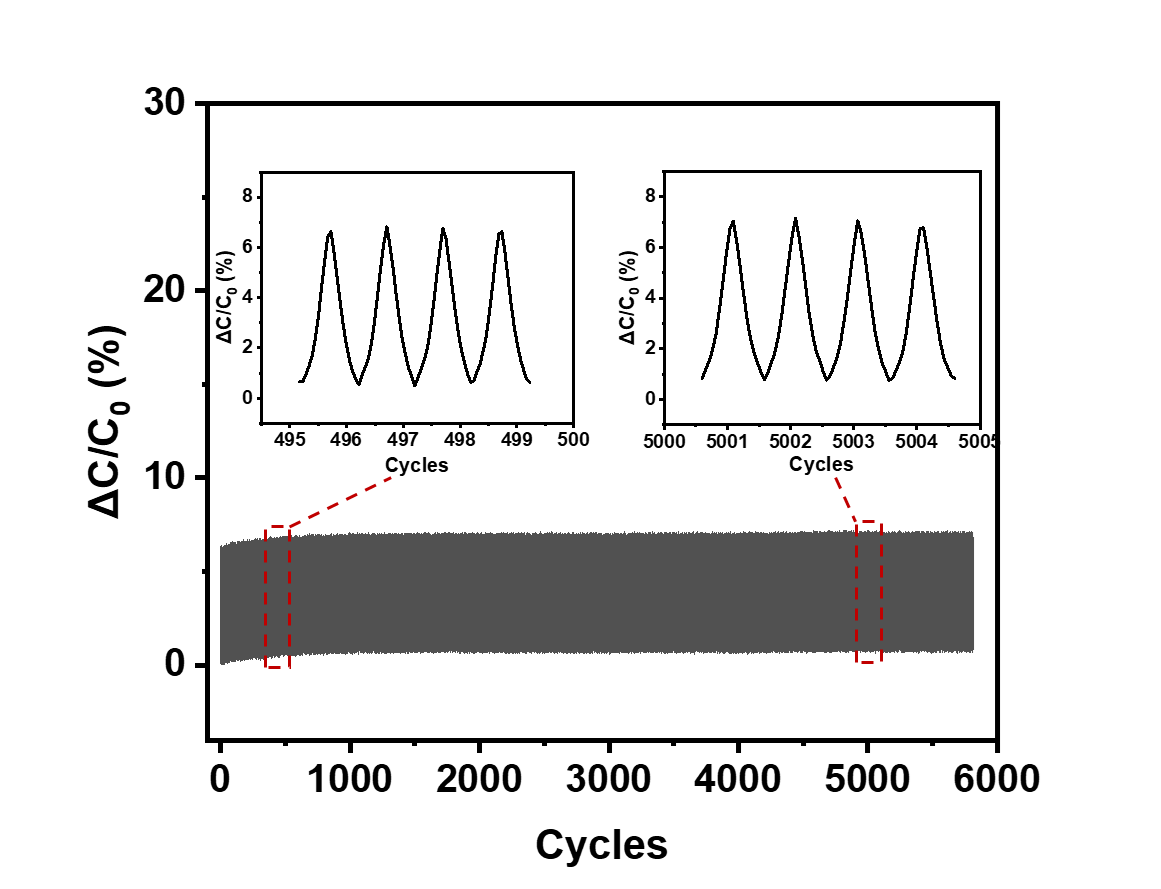


Figure S17. Cyclic capacitive data of original one. Stable operation over 5800 cycles.

| **Electrode / Dielectric material** | **Pressure Range (kPa)** | **Sensitivity (**$\mathbf{kPa}^{\boldsymbol{-1}}$**)** | **Detection Limit (Pa)** | **Ref** |
| --- | --- | --- | --- | --- |
| Copper electrode / P₄-*^t^*Bu reconfigured silicone | 0 - 5  5 – 160 | 4.67  0.14 | 8.12 | Our work |
| Nickel fabric electrode / 3D hierarchical spacer fabric | 0 - 110 | 0.33 | 0.25 | [1] |
| Aluminum fabric / porous PDMS | 0 - 8 | 4.99 | - | [2] |
| Gold-plated copper pad electrodes / BTO NWs -TPU porous composites film | 0 - 10 | 1.47 | - | [3] |
| Silver paste /  Carbon nanotubes-silicone rubber | 0 – 4.5 | 0.062 | 3 | [4] |
| Titane and gold electrode / Polydimethylsiloxane micro-pyramids | 0 – 1  0.75 – 2.5 | 0.1  0.05 | 20 | [5] |
| Copper electrode / MXene, MWCNTs, PDMS composite | 0.005 - 50 | 0.32 | 0.18 | [6] |
| Copper electrodes / Gallium microgranules embedded in PDMS | 0 – 1  1-1450 | 16.97  0.02 | 3 | [7] |

Table S1. Comparative material performance of flexible capacitive pressure sensors.

**Note S1.**

Table S1 presents a detailed comparison of the sensor’s key performances in relation to state-of-the-art soft, flexible capacitive pressure sensors including: the detection range, detection limit and sensitivity.

To increase the performance of the dielectric layer in flexible capacitive pressure sensors, different approaches have been used in literature, such as using alternative materials to PDMS (fabric [1]), micro structuring (pyramidal or porous architectures [2,5]), or filler-doping strategies (carbon nanotubes or gallium microgranules in PDMS [4,7]). Our device stands out by a simple and scalable approach, by modifying a commercially available silicone matrix with a catalyst, making the fabrication process more accessible while maintaining strong and competitive performance across multiple metrics.

*Performance Breakdown:*

1. *Pressure Range, Resolution and Sensitivity:* Our sensor operates across a broad pressure range, covering both ultra-low and moderately high pressures. In the high-sensitivity regime (0.008–5 kPa), it achieves a sensitivity of 4.67 kPa⁻¹, while in the higher-pressure range (5–160 kPa), it maintains a sensitivity of 0.14 kPa⁻¹. For comparison, Jeong et al. [7] reported a pressure sensor with a very high sensitivity of 16.97 kPa⁻¹ in the <1 kPa range, but it exhibited a significantly lower sensitivity of 0.02 kPa⁻¹ at higher pressures. This dual-regime performance allows our sensor to surpass existing devices by offering both high sensitivity and an extended detection range, as summarized in Table S1. The combination of broad pressure coverage, stable response, and minimal hysteresis ensures reliable and versatile operation across a wide spectrum of soft robotic and wearable sensing applications.
2. *Detection Limit and Pressure Resolution:* We experimentally determined a detection limit of 8.12 Pa using the setup described in the Experimental Section. Figure S16 (Supplementary Information) shows the sensor’s response to a sinusoidal pressure input between 0 and 8.12 Pa. Pressures below this threshold result in signal-to-noise ratios too low for reliable detection. Therefore, both the detection limit and effective pressure resolution are established at 8 Pa. Different approaches presented in Table S1 such as doping PDMS with carbon nanotubes enable even smaller detection limits (<3Pa [3,6]) but result in lower sensitivity and pressure range.

[1] Ye, X., Shi, B., Li, M., Fan, Q., Qi, X., Liu, X., Zhao, S., Jiang, L., Zhang, X., Fu, K. and Qu, L., 2022. All-textile sensors for boxing punch force and velocity detection. *Nano Energy*, *97*, p.107114.

[2] Kim, Y., Yang, H. and Oh, J.H., 2021. Simple fabrication of highly sensitive capacitive pressure sensors using a porous dielectric layer with cone-shaped patterns. *Materials & Design*, *197*, p.109203.

[3] Nie, L., Zhang, L., Di, X., Liu, Q., Zhang, Z.A., Zhou, Q., Dong, Z., Song, Z., Zhang, S. and Pan, G., 2022. Assembly of highly-sensitive capacitive flexible pressure sensor based on BTO NWs-TPU porous composites film. *Vacuum*, *205*, p.111423.

[4] Qiu, J., Guo, X., Chu, R., Wang, S., Zeng, W., Qu, L., Zhao, Y., Yan, F. and Xing, G., 2019. Rapid-response, low detection limit, and high-sensitivity capacitive flexible tactile sensor based on three-dimensional porous dielectric layer for wearable electronic skin. *ACS applied materials & interfaces*, *11*(43), pp.40716-40725.

[5] Thouti, E., Nagaraju, A., Chandran, A., Prakash, P.V.B.S.S., Shivanarayanamurthy, P., Lal, B., Kumar, P., Kothari, P. and Panwar, D., 2020. Tunable flexible capacitive pressure sensors using arrangement of polydimethylsiloxane micro-pyramids for bio-signal monitoring. *Sensors and Actuators A: Physical*, 2020, vol. 314, p. 112251.

[6] Li, X., Liang, Q., Liu, H., Zhao, L., Sun, C. and Hou, C., 2025. High‐Sensitivity MXene/MWCNTs/PDMS Flexible Capacitive Sensor for Wearable Health Monitoring. *Advanced Materials Technologies*, S. e00677.

[7] Lee, S., Byun, S.-H., Kim, C.Y., Cho, S., Park, S., Sim, J.Y. and Jeong, J.-W., 2022. Beyond human touch perception: An adaptive robotic skin based on gallium microgranules for pressure sensory augmentation. *Advanced Materials, 34(44), 2204805.*
